# Robust metric for quantifying the importance of stochastic effects on nanoparticle growth

**DOI:** 10.1038/s41598-018-32610-z

**Published:** 2018-09-21

**Authors:** Tinja Olenius, Lukas Pichelstorfer, Dominik Stolzenburg, Paul M. Winkler, Kari E. J. Lehtinen, Ilona Riipinen

**Affiliations:** 1 Department of Environmental Science and Analytical Chemistry (ACES) and Bolin Centre for Climate Research, Stockholm University, SE-10691 Stockholm, Sweden; 2 Division of Physics and Biophysics, Department of Materials Research and Physics, University of Salzburg, A-5020 Salzburg, Austria; 3 Faculty of Physics, University of Vienna, A-1090 Vienna, Austria; 4 Department of Applied Physics, University of Eastern Finland and Finnish Meteorological Institute, POB 1627, FI-70211 Kuopio, Finland

## Abstract

Comprehensive representation of nanoparticle dynamics is necessary for understanding nucleation and growth phenomena. This is critical in atmospheric physics, as airborne particles formed from vapors have significant but highly uncertain effects on climate. While the vapor–particle mass exchange driving particle growth can be described by a macroscopic, continuous substance for large enough particles, the growth dynamics of the smallest nanoparticles involve stochastic fluctuations in particle size due to discrete molecular collision and decay processes. To date, there have been no generalizable methods for quantifying the particle size regime where the discrete effects become negligible and condensation models can be applied. By discrete simulations of sub-10 nm particle populations, we demonstrate the importance of stochastic effects in the nanometer size range. We derive a novel, theory-based, simple and robust metric for identifying the exact sizes where these effects cannot be omitted for arbitrary molecular systems. The presented metric, based on examining the second- and first-order derivatives of the particle size distribution function, is directly applicable to experimental size distribution data. This tool enables quantifying the onset of condensational growth without prior information on the properties of the vapors and particles, thus allowing robust experimental resolving of nanoparticle formation physics.

## Introduction

Understanding the dynamics of nanoparticle populations is essential for probing nucleation, coalescence processes and phase transitions in various fields of fluid mechanics, soft matter physics and geosciences. Nanoparticle formation from condensable vapors is also a frequent phenomenon in the Earth’s atmosphere^1^. A substantial fraction of all airborne aerosol particles are estimated to originate from such gas-to-particle conversion^2–5^, but these estimates are highly sensitive to assumptions on (1) the formation mechanisms in different environments, and (2) the dynamics of the smallest nanoparticles (<5–10 nm in diameter)^5,6^. These questions are of central importance for the advancement of atmospheric physics: besides being a key component of air quality, aerosol particles have a potentially large, although highly uncertain, impact on clouds and climate^7,8^. As the smallest nanoparticles are easily lost from the air by removal processes, the early growth dynamics is a crucially important factor affecting particle survival to larger, climatically relevant sizes.

During the recent decade, experimental techniques measuring airborne nanoparticle concentrations down to the smallest molecular clusters of diameters of ca. 1–2 nm have been developed and deployed in laboratory and field^9–14^. While this is an important step forward, interpreting these observations is difficult due to unknown properties of the vapors and particles, namely the rate constants of the molecular collision and attachment (i.e. condensation), evaporation and coagulation processes. Evaporation rate constants, determined by the complex thermochemistry of the small particles, are the most challenging parameters to quantify, with uncertainties spanning up to orders of magnitude^15–18^.

Theoretical treatment of nanoparticle dynamics can be divided into (1) modeling the initial clustering with molecule-by-molecule models, and (2) describing the subsequent condensational growth assuming a macroscopic, continuous substance omitting stochastic collisions and evaporations of single molecules^19,20^. The initial cluster formation can occur via nucleation or barrierless clustering. In the former case, the particle evaporation frequency exceeds the collision frequency with vapor molecules at the smallest sizes, and stochastic fluctuations in particle size drive the growth until the collisions overcome evaporation at the critical size region^19,21^. Stochastic effects are likely non-negligible at the smallest sizes also for barrierless, collision-driven clustering^22^. However, due to the poorly known rate constants there has been no direct way to determine the particle sizes at which these effects become negligible. With no accurate knowledge on this limiting size range, experimentally observed size distributions are typically analyzed using continuous modeling frameworks from particle diameters of ca. 1–2 nm onward^23–27^. The validity of this assumption and the related errors have not been quantitatively addressed to date.

Reliably constraining the rate constants controlling observed nanoparticle formation phenomena is necessary for resolving the detailed physics and chemistry behind the process, and for predicting the size-dependent particle number. Assessing these parameters from experiments requires further development of sophisticated inverse modeling approaches^26,28^, and the first step for this is determining which type of physical model is suitable for the studied particle size range. The fundamental molecule-by-molecule approach cannot be expanded to very large sizes due to its vast computational burden and complexity, which increase drastically with increasing particle diameter. Accurately determining the threshold size for continuum growth is a key question, as it allows extending the simpler and computationally efficient continuous description down to as small sizes as possible. Here we present a simple, robust, and generalizable metric for quantifying the importance of stochastic vs. deterministic effects on nanoparticle populations, based on theoretical considerations of population dynamics. Simulations and experimental data in sub-10 nm size range confirm the validity and applicability of the approach. We show that the shape of the nanoparticle size distribution indicates the size regime below which stochastic effects cannot be omitted, with no need for prior knowledge of the related rate constants. Finally, we discuss the implications for interpretation of measurements and for prediction of airborne particle concentrations.

## Results

### Discrete and continuous descriptions of nanoparticle dynamics

The dynamics of an evolving nanoparticle population are fundamentally described by the discrete general dynamic equation (GDE)

1 $$\frac{{\rm{d}}{C}_{i}}{{\rm{d}}t}=\frac{1}{2}\sum _{j < i}({\beta }_{j,i-j}{C}_{j}{C}_{i-j}-{\gamma }_{i\to j,i-j}{C}_{i})-\sum _{j}({\beta }_{i,j}{C}_{i}{C}_{j}-{\gamma }_{i+j\to i,j}{C}_{i+j})+{Q}_{i}-{S}_{i}{C}_{i}.$$

Eq. (1) gives the time derivative of the number concentration *C*_*i*_ of particle *i* of a given molecular composition including all condensation, evaporation, particle coagulation and removal processes. The first summation includes molecular and coagulational collisions forming particle *i*, and the corresponding evaporations destroying it; the second summation corresponds to particle *i* colliding with vapor molecules and other particles *j*, and to evaporations resulting back to particle *i*. *β*_*i*,*j*_ and *γ*_*i*+*j* → *i*,*j*_ are the collision and evaporation rate constants, respectively. The source term *Q*_*i*_ normally applies only to vapor molecules, and the size-dependent sink rate constant *S*_*i*_ to all molecules and particles. Generally, evaporation of only single vapor molecules is considered, as fissions are expected to be rare. Coagulation is negligible when particle concentrations are significantly lower than vapor concentrations, but becomes important when particle concentrations are increased due to high vapor sources, low sinks and/or suppressed evaporation.

The continuous form of the GDE is derived by transforming the concentration of discrete particle sizes into a continuous function of particle size and time. While the coagulation and removal terms of the continuous GDE are analogous to the discrete presentation, the condensation–evaporation terms are essentially different. In the discrete GDE, the attachment and evaporation of vapor molecules is described as

2 $${(\frac{{\rm{d}}{C}_{i}}{{\rm{d}}t})}_{{\rm{cond}}}={\beta }_{1,i-1}{C}_{1}{C}_{i-1}-{\gamma }_{i\to 1,i-1}{C}_{i}-{\beta }_{1,i}{C}_{1}{C}_{i}+{\gamma }_{i+1\to 1,i}{C}_{i+1},$$

where subscript 1 refers to a single molecule. The continuous form of Eq. (2) is obtained via a Taylor expansion of *C*, *β* and *γ* around size *i*^29,30^. Including derivatives up to the second order gives the Fokker-Planck equation

3 $${(\frac{\partial c}{\partial t})}_{{\rm{cond}}}=-\,\frac{\partial }{\partial i}[(\beta {C}_{1}-\gamma )c]+\frac{1}{2}\frac{{\partial }^{2}}{\partial {i}^{2}}[(\beta {C}_{1}+\gamma )c],$$

where the continuous function *c*(*i*, *t*) is the concentration density per size interval. The first-order term, also called the drift term, describes the deterministic particle growth, governed by the driving force of condensation ∝ (*βC*_1_ − *γ*). The second-order term corresponds to diffusion in particle size space, driven by the stochastic molecular collisions and evaporations. Omitting the second-order term in Eq. (3) gives the standard continuous form, henceforth referred to as the continuous condensation description

4 $${(\frac{\partial c}{\partial t})}_{{\rm{cond}}}=-\,\frac{\partial }{\partial i}[(\beta {C}_{1}-\gamma )c].$$

A fundamental property of the continuous condensation equation is that it does not include stochastic effects: in Eq. (4), all particles of a given size *i* grow or shrink according to frequency *βC*_1_ − *γ*, and an initially monodisperse distribution always remains monodisperse. By contrast, the discrete condensation equation (Eq. (2)) and the Fokker-Planck equation (Eq. (3)) allow the stochastic widening of the size distribution, and describe both diffusion-driven nucleation and drift-driven growth.

As the studied particle size range increases, the GDE is more conveniently presented via particle diameter *d*_p_, and the distribution is described by the concentration density per diameter interval *c*′ = *c* × d*i*/d*d*_p_. The condensational growth equation (Eq. (4)) becomes

5 $${(\frac{\partial c^{\prime} }{\partial t})}_{{\rm{cond}}}=-\,\frac{\partial }{\partial {d}_{{\rm{p}}}}({{\rm{GR}}}_{{\rm{cond}}}c^{\prime} ),$$

where GR_cond_ is the change rate of the particle diameter when stochastic effects are omitted. For an arbitrary number of condensing vapor species,

6 $${{\rm{GR}}}_{{\rm{cond}}}=\frac{{\rm{d}}{d}_{{\rm{p}}}}{{\rm{d}}t}=\frac{{\rm{d}}{d}_{{\rm{p}}}}{{\rm{d}}{m}_{{\rm{p}}}}\frac{{\rm{d}}{m}_{{\rm{p}}}}{{\rm{d}}t}=\frac{2}{\pi {\rho }_{{\rm{p}}}{d}_{{\rm{p}}}^{2}}\sum _{k}({\beta }_{k}{C}_{k}-{\gamma }_{k}){m}_{k},$$

where *m*_p_ and *ρ*_p_ are the mass and density of the particle of diameter *d*_p_, and the summation goes over the mass fluxes of vapors *k* of mass *m*_*k*_. Due to its apparent link to large-scale modeling and the thermochemical properties of the vapors, Eq. (6) is one of the key approaches used to interpret experimentally observed nanoparticle formation^19,20,24,25^. Coagulation and scavenging of particles by external surfaces, such as large aerosols in the atmosphere and chamber walls in the laboratory, can be accounted for when assessing GR_cond_ from observations by applying the full GDE^23,31^. A fraction of vapors may also be bound to clusters of a few molecules, and the contribution of these clusters to the growth of larger particles can be included in GR_cond_.

### Stochastic vs. deterministic effects on condensational growth

Here we simulate nanoparticle formation in sub-10 nm size regime by solving the discrete GDE including condensation, evaporation, coagulation and particle sinks (Eq. (1); see Methods). Possible particle-phase processes affecting particle chemistry are not included. We focus on situations where nucleation, condensation and evaporation are the main processes affecting particle formation, but include also cases where coagulation becomes significant. We use the discrete simulation data to evaluate standard data analysis approaches based on assuming continuous condensational growth. The default simulation conditions correspond to a chamber experiment^26,32^, and the molecules are representative of oxidized low-volatile organic compounds (LVOC), which are recognized as a major driver of atmospheric nanoparticle growth^24,26,33–35^. Complementary simulations are conducted including an extremely low-volatile compound (ELVOC). To treat the simulated particle concentrations similarly to measurable quantities, particles are grouped in size bins according to their mobility diameter, defined as *d*_p,mass_ + 0.3 nm where *d*_p,mass_ is the mass diameter^36^, with a bin width of 0.1 nm. Other measurement non-idealities, such as size-dependent detection efficiency and instrumental noise, are assumed to be corrected for.

To verify that the conclusions are independent of the simulation rate constants, additional simulations are performed using different compound properties and ambient conditions, and qualitatively and quantitatively different particle evaporation rates. The evaporation rates have a large impact on the size distribution dynamics, but quantifying these rates is extremely challenging: the classical Kelvin formula (Supplementary Eq. (S1)) is expected to give a qualitatively reasonable size dependence, as small molecular clusters are generally more prone to evaporation than larger nanoparticles due to their larger surface-to-volume ratio. However, the thermochemistry of these small complexes is affected by atom-scale phenomena such as the degree and patterns of hydrogen bonding and proton transfers, which are not expected to be similar to liquids and larger particles. The Kelvin formula is thus not considered to give accurate results for the smallest particle sizes. The most accurate method to assess the properties of small clusters is quantum chemistry^37^, but even the best quantum chemical methods involve high uncertainties stemming from, for instance, limitations in capturing the electron correlation especially for clusters of more than a couple of molecules. These issues may propagate to uncertainties of more than an order of magnitude in the evaporation rates^15,16^. Moreover, the available quantum chemical data is mainly for sulfuric acid and inorganic or organic basic species; clusters containing several oxidized organic molecules are too heavy for the current capacity of the methods. Therefore, we apply different evaporation rate profiles of a realistic order of magnitude: the evaporation rates are either approximated with the Kelvin formula (Supplementary Eq. (S1)), set to vary randomly while decreasing with particle size (Supplementary Eq. (S2)), or calculated from quantum chemical data for tests with representative acid–base systems. Details of all simulation set-ups and additional discussion are found in Supplementary Information.

**Figure 1:**
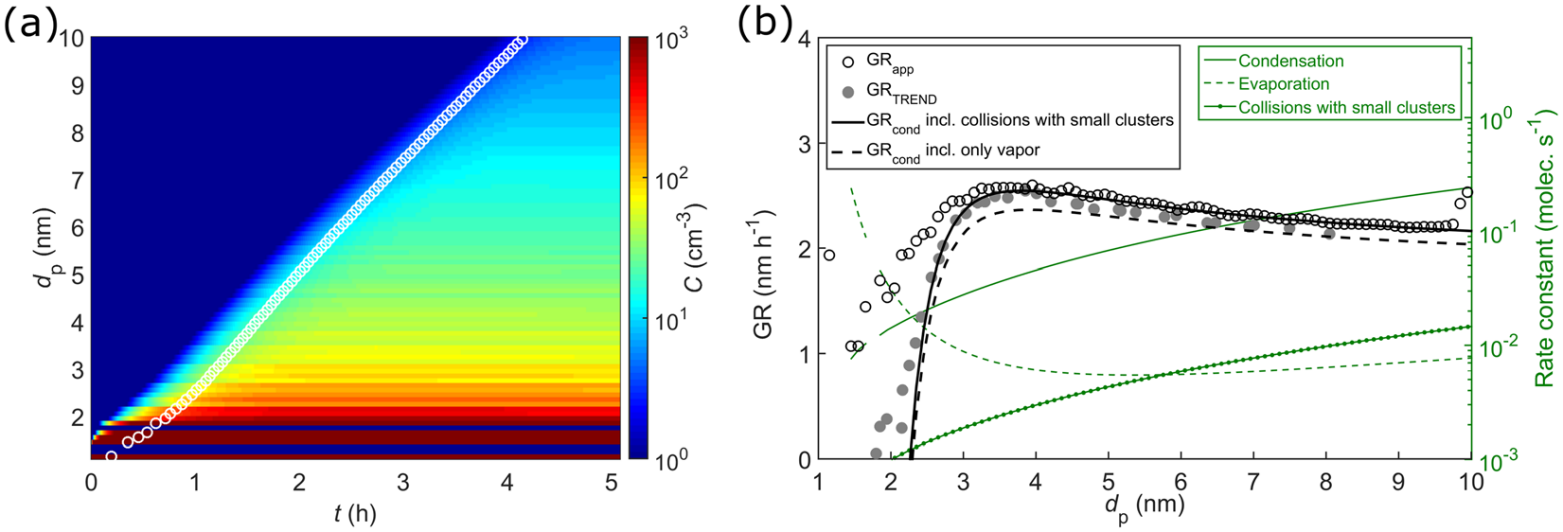
Panel (a): Simulated nanoparticle formation event at conditions of a chamber experiment for a representative LVOC species at a final vapor concentration of *C*_LVOC_ = 2∙10^7^ cm^−3^. White circles depict size bin appearance times *t*_app_. Panel (b): GR_app_ determined from *t*_app_ (open circles; Eq. (7)), GR_TREND_ determined by the TREND method (filled circles), and the condensational growth rate GR_cond_ (Eq. (6)) with and without including collisions with very small clusters (solid and dashed black lines, respectively). The collision and evaporation frequencies from which GR_cond_ is calculated are shown on the right-hand side y-axis.

Figure 1a demonstrates the standard experimental analysis approach^26,38,39^. A vapor source is turned on in a laboratory chamber, and the appearance of subsequent particle sizes is observed as the size distribution builds up. Since the initial particle sizes do not form a clear growing mode, methods based on following the growth of such a mode^40^ cannot be used. Instead, each size bin *d*_p_ is assigned an appearance time *t*_app_ at which the concentration in the bin reaches 50% of its maximum value. The apparent growth rate GR_app_ is defined as the slope of the (*t*_app_, *d*_p_)-data

7 $${{\rm{GR}}}_{{\rm{app}}}\equiv \frac{{\rm{\Delta }}{d}_{{\rm{p}}}}{{\rm{\Delta }}{t}_{{\rm{app}}}}.$$

This is compared to the continuous-GDE-based condensational growth rate GR_cond_ (Eq. (6)), which here includes also clusters of a couple of molecules (see Methods), because in some simulation cases they may make a minor contribution to GR_app_ (see Fig. 1b). Figure 1b shows that at larger sizes (here *d*_p_ ≳ 3 nm), GR_app_ approaches GR_cond_, but it is evident that at the smallest end of the size spectrum, GR_app_ and GR_cond_ differ drastically as stochastics causes a fraction of particles of a given size to grow faster than the average rate GR_cond_. Specifically, in case of genuine nucleation where the first sizes are unstable against evaporation (here *d*_p_ ≲ 2.3 nm), GR_cond_ is negative for the initial sizes and approaches GR_app_ from below as the size increases. Predictions of condensation calculations can thus be expected to be inherently lower than the observed growth at the small end.

On the other hand, while the appearance-time-based method has become an established analysis approach, extracting growth rates from observations is not unambiguous. This applies especially to conditions at which particle sinks and coagulation have prominent effects on the distribution^41^. To confirm the conclusions, a recently developed growth rate analysis tool TREND^31^ that accounts for these effects was also applied. TREND determines the size- and time-resolved condensational growth rates by comparing regions of measured (here synthetic) and modeled particle size distributions (see Methods). The TREND results, also presented in Fig. 1b, show that also GR_TREND_ is indeed higher than GR_cond_ at the build-up of the initial sizes, similarly to GR_app_.

### Metric for determining the importance of stochastic effects

In real experiments, GR_cond_ cannot be readily calculated due to uncertainties related to the properties and detection of various types of vapors^17,18,26,27^. However, fitting GR_cond_ to reproduce GR_app_ outside the validity range of the continuous model (in Fig. 1b, below ca. 3 nm) results in erroneous conclusions on the condensational growth mechanisms. As stochastic effects are described by the second-derivative term in the Fokker-Planck equation (Eq. (3)), we propose that the first and second derivatives of the distribution *c*(*i*) or *c*′(*d*_p_) can be used to assess the sizes starting from which observed growth can be interpreted omitting stochastics. In Eq. (3), the derivatives are taken of fluxes, i.e. include both the particle concentration *c* and the rate constants *βC*_1_ and *γ*. While only *c* can be directly observed, a strong size-dependence in the rate constants is expected to propagate to a strong size-dependence in the concentration, and thus we hypothesize that studying the gradients of the distribution gives information on the size-diffusion effects (see also Supplementary Information Section 1.4).

**Figure 2:**
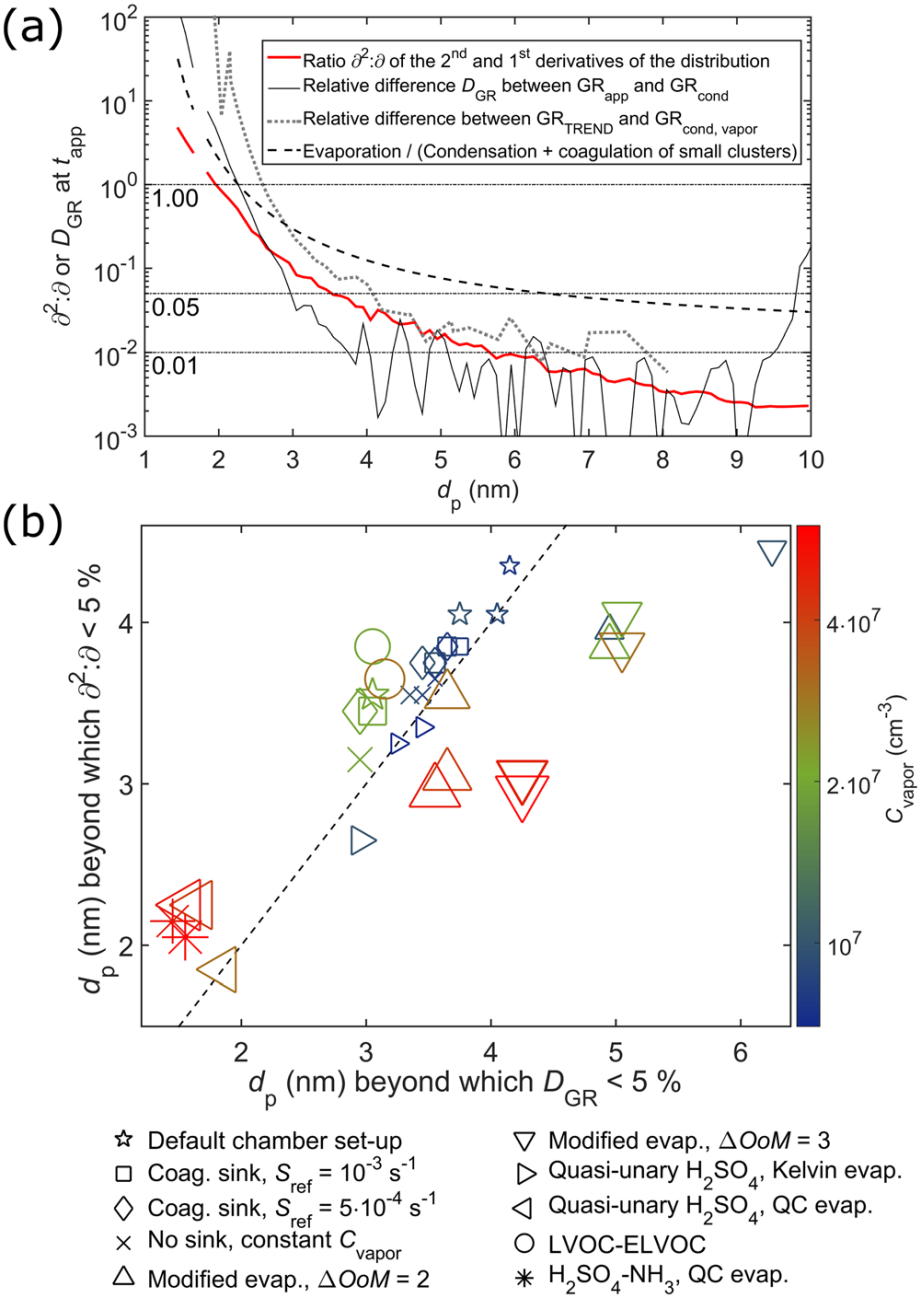
Panel (a): Relative difference *D*_GR_ = abs[(GR_cond_ − GR_app_)/GR_app_] between GR_app_ and GR_cond_ (thin solid line), and the ratio *∂*^2^:*∂* of the second and first derivatives of the distribution at *t*_app_ (thick solid line) for LVOC at *C*_LVOC_ = 2∙10^7^ cm^−3^. Dotted grey line shows the difference between GR_TREND_ and GR_cond,vapor_, where GR_cond,vapor_ includes only vapor (see Methods). Black dashed line shows the particle stability as the ratio of the evaporation and condensation rates. Panel (b): The size at which GR_app_ and GR_cond_ converge within 5%, and the size at which *∂*^2^:*∂* falls below 5% for different simulation cases (see Supplementary Information). The color and size of the markers depict the final vapor concentration *C*_vapor_.

Figure 2a shows the relative difference *D*_GR_ between GR_app_ and GR_cond_ together with the ratio of the second and first derivatives of the distribution (see Methods)

8 $$\frac{{\partial }^{2}c}{\partial {i}^{2}}/\frac{\partial c}{\partial i}\equiv {\partial }^{2}:\,\partial .$$

The differences between GR_app_ and GR_cond_ become negligible at the sizes at which *∂*^2^:*∂* drops to a few percent. Furthermore, *D*_GR_ and *∂* ^2^:*∂* are generally of similar magnitude around the size of convergence, tentatively suggesting that *∂* ^2^:*∂* gives a rough estimate of the magnitude of the error in GR_cond_ around this size. Figure 2b compares the size around which GR_app_ and GR_cond_ converge and the size around which *∂*^2^:*∂* becomes negligible for different simulation cases covering a variety of rate constant profiles and set-ups. The comparison is striking: the data falls around a 1:1 line, indicating that *∂*^2^:*∂* can be reliably used as a metric to quantify the limits of the continuum model.

The size range where GR_app_ and GR_cond_ converge is largely affected by particle stability, which is depicted by the ratio of the evaporation and condensation frequencies in Fig. 2a. As the vapor concentration increases, the critical size region at which collisions overcome evaporation shifts towards smaller sizes. Since growth through stochastic collisions is more important when evaporation is relatively significant, also the convergence size of GR_app_ and GR_cond_ becomes smaller at higher vapor concentrations (see data points corresponding to same set-up (symbol) but different vapor concentration (color and size) in Fig. 2b). Therefore, the *∂*^2^:*∂* analysis can be used to roughly fork the critical regime of clustering, which is connected to the overall thermodynamics of the initial particle formation^20^. However, *D*_GR_ and *∂*^2^:*∂* are also affected by external conditions: the size distribution becomes steeper with increasing particle sink, shifting the convergence region towards slightly larger sizes (cf. the sink-free case (crosses) and the sink cases (diamonds, squares, and stars, in order of increasing sink) in Fig. 2b). In general, in addition to stochastics-driven growth, the early evolution of the distribution and the appearance of the smallest sizes may be significantly affected by particle sinks and vapor sources (see Supplementary Discussion Section 2.4). Simulations with evaporation rates modified to vary randomly around the values given by Supplementary Eq. (S1), or based on quantum chemistry, exhibit the same decreasing trend with respect to vapor concentration, but may differ somewhat more from the 1:1 line. This is due to non-smooth evaporation profiles, which cause larger fluctuations in *D*_GR_ and to some extent also in *∂*^2^:*∂*.

**Figure 3:**
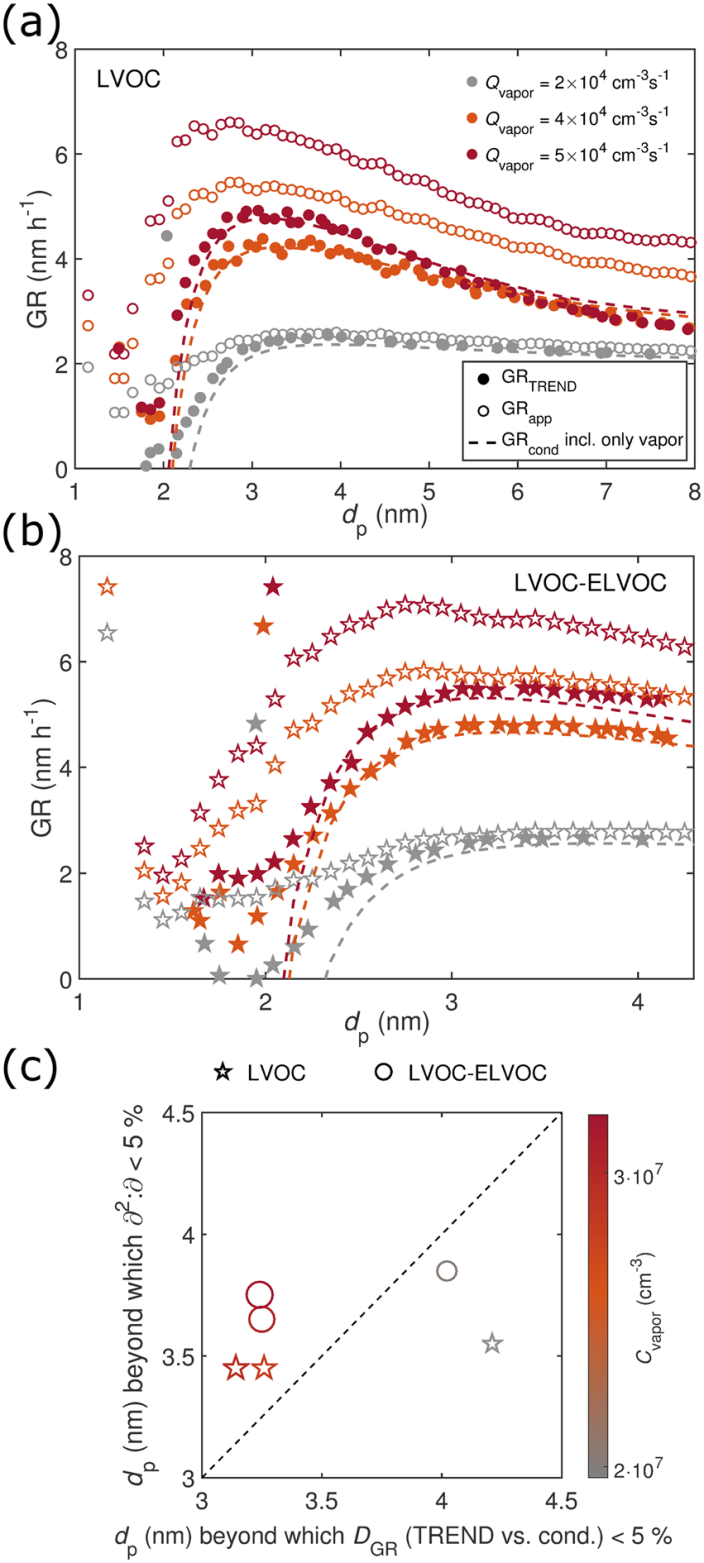
Panels (a) and (b): GR_cond,vapor_, GR_TREND_, and GR_app_ at particle appearance times *t*_app_ for LVOC and LVOC–ELVOC, respectively. Panel (c): The size at which GR_TREND_ and GR_cond,vapor_ converge within 5%, and the size at which *∂*^2^:*∂* falls below 5%. Note that GR_cond,vapor_ is calculated here considering only single vapor molecules to be consistent with GR_TREND_.

As GR_app_ does not allow separating the contribution of coagulation among the population, cases where coagulation becomes significant were examined with TREND as shown in Fig. 3 (see also Supplementary Discussion Section 2.2). In general, these include high vapor sources and the presence of strongly clustering compounds (here ELVOC), which lead to elevated particle concentrations. Regardless of coagulation, the general results are similar to GR_app_: the condensational growth rate is distorted for the initial sizes (panels (a) and (b)), and the convergence size is smaller at higher vapor levels (panel (c)). TREND does not, however, give as high values for the small sizes as the appearance time method.

It must be emphasized that the reasoning behind the metric *∂*^2^:*∂* is independent of the values and size dependences of the collision and evaporation rate constants *β* and *γ*. The rate constants of different dynamic processes shape the particle size distribution, creating gradients to the size-dependent concentration. If there is a strong size-dependence in the derivatives of the fluxes *βC*_1_*c* and *γc* between consecutive particle sizes (Eq. (3)), the simplified condensation equation (Eqs (4) and (5)) is not valid (see also Supplementary Information Section 1.4). Therefore, applying *∂*^2^:*∂* does not require prior knowledge of the rate constants, or of the physical and chemical processes affecting them. Due to its general considerations, the metric applies to different types of particle formation events and methods to deduce growth rates. This includes also e.g. the growth of a mode involving a seemingly sharp peak in the distribution. Even if a peak is distinct in terms of particle diameter, the growth can be described by continuous condensation if the second-order derivative around the peak is small in terms of molecular additions (Eqs (2, 3 and 11)). Finally, for the standard appearance time method, it can be noted that *∂*^2^:*∂* at each size is evaluated here at *t*_app_, at which the bin reaches 50% of its maximum concentration. The growth rates and *∂*^2^:*∂* are, however, time-dependent, and thus *D*_GR_ can vary with time (Supplementary Information Sections 2.2 and 2.3). Also other definitions of appearance time have been used, and Supplementary Fig. S8 demonstrates that *D*_GR_ increases with decreasing threshold concentration for determining *t*_app_. This is because the gradient *∂c*/*∂i* varies more strongly at the beginning of the formation event.

### Applying the metric *∂*^2^:*∂* on experimental data

The comprehensive set of test simulations was used to determine how to robustly capture the shape of a given particle size distribution *c*′(*d*_p_) and to obtain the metric *∂*^2^:*∂*. Imperfect size resolution leads to a less smoothly behaving distribution, and the distribution may take different shapes depending on the conditions. The results indicate that an observed distribution can be used to quantify the size regime where particle growth mechanisms shift from stochastics-influenced clustering to deterministic, mass-flux-driven condensation by determining *∂*^2^:*∂* as follows (see Methods):

1. The size resolution at nanometer sizes needs to be fine enough. For the modeled molecule types, the resolution must be at least approximately 1.0 nm, but preferably higher.
2. The 1^st^ and 2^nd^ derivatives of the distribution *c*′(*d*_p_) with respect to particle diameter *d*_p_ can be obtained as analytical derivatives of a 3^rd^ order polynomial fit on the concentration, adjusting the fitted size range so that the function reliably captures the shape and gradients of the particle concentration. This was achieved using approximately ten adjacent data points for the model data.
3. Finally, *∂*^2^:*∂* is obtained from the 1^st^ and 2^nd^ derivatives by Eq. (11). This requires an estimate of the average molecular volume, but the results are not very sensitive to the accuracy of this estimate.

**Figure 4:**
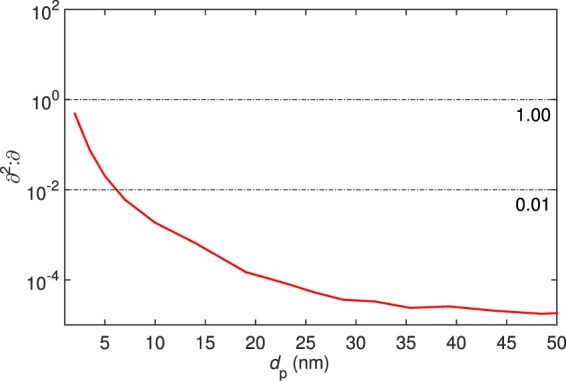
∂^2^:∂ determined for an experimentally observed size distribution (Fig. 3a in ref.^31^) of nanoparticles formed from organic compounds from α-pinene oxidation.

Figure 4 presents *∂*^2^:*∂* determined for an experimentally measured size distribution for particle formation from α-pinene oxidation products at the aerosol chamber of National Center for Atmospheric Research (NCAR)^31^. The metric exhibits a trend strikingly similar to the synthetic data: *∂*^2^:*∂* falls below a few percent at ca. 3–5 nm, indicating the onset of drift-driven condensational growth. While the chemical properties of the compounds present at the experiment remain to be quantified, Fig. 4 suggests to apply continuous-GDE-based models for sizes from ca. 5 nm upward for reliably resolving the particle growth mechanisms.

## Discussion

The results raise important points regarding the interpretation of observations of very small particles. While continuous condensation models serve as a suitable first-order approximation, their limits and uncertainties have remained unquantified to date. The smallest particles require a discrete, molecule-by-molecule treatment^32,42–44^, and applying the continuous model outside of its validity range can lead to serious misinterpretations of observation data. However, extending the computationally efficient continuous description down to its lower limits is necessary due to the enormous computational burden of discrete modeling. For a mixture of vapors, the number of coupled differential equations in a discrete model rapidly increases to thousands and beyond even in the sub-5 nm size range. Finding an optimal and robust modeling approach is required for systematic and reliable assessment of particle evaporation rates and other key parameters from measured particle concentrations. This analysis is necessary for predicting the number and size distribution of newly-formed nanoparticles and their response to changes in ambient conditions. Correct modeling of the growth processes is relevant also for measurement techniques, e.g. for assessing the activation of particles to condensational growth inside condensation particle counters.

The presented results highlight the importance of accurately determining the threshold size for continuum approaches: fitting a deterministic condensation model to reproduce the observed apparent growth in situations where stochastics play a major role can lead to erroneous conclusions on (see e.g. the data in Fig. 1b) (1) the thermodynamic and other properties of the vapors and particles (when adjusting e.g. the Kelvin formula to match given data), (2) “missing” condensing species (the stochastic growth rate may be significantly higher than the deterministic prediction), and (3) the presence and magnitude of a Kelvin barrier at very small sizes^26,38^. The time evolution of the population at the initial sizes may be largely determined by stochastics-driven processes, particle sinks and the time dependence of vapor concentrations, and thus the size dependence of the apparent condensational growth rate is not necessarily related to particles growing past thermodynamic barriers. While the experimental growth rate may be quantified differently by different data analysis methods, these issues occur regardless of the method used. This is demonstrated e.g. in ref.^41^ by applying different methods to synthetic particle population data in the nanometer size range. Finally, the apparent growth may include also coagulation effects at elevated nanoparticle concentrations^41,45^. These need to be accounted for^31^, but the issue of stochastics vs. deterministic contributions on the growth due to vapor–particle exchange applies also in this case.

Within atmospheric sciences, correct representation of the initial growth is important not only for understanding local-scale particle pollution, but also for predictions of aerosol–cloud interactions which continue to be the single largest source of uncertainty in assessments of Earth’s radiation budget and global warming^8^. During atmospheric aerosol formation, small particles are lost to scavenging sinks due to their high mobility, but the loss rate decreases rapidly with increasing particle size. The early growth dynamics below ca. 5–10 nm are critical for aerosol number and size distribution, as faster growth leads to more particles reaching larger sizes^20,46^. The number of particles growing to ca. 50–100 nm, at which they can act as cloud condensation nuclei (CCN), is essential for the formation and properties of clouds. In large-scale models, production of particles of a few nanometers (often ≳ 3 nm) is commonly approximated based on assumed condensational growth by scaling the initial particle formation rate (at ca. 1 nm) by an exponential factor depending on the particle growth and loss rates^6,46,47^. At typical conditions, an overestimation of e.g. a factor of 2–5 in the growth rate of 1–3 nm particles results in an overestimation of a factor between ca. 2 and >>10 in the formation rate of >3 nm particles (see also Supplementary Discussion Section 2.5). The importance of these early growth stages on global aerosol and CCN concentrations has been demonstrated e.g. in ref.^26^ by atmospheric simulations assuming different parameterizations for the growth rate in the 1.7–3 nm size range: changing the parameterization resulted in up to 50% changes in the CCN concentrations. Misinterpretation of the apparent growth rate from e.g. laboratory data may thus lead to distorted assessments of the number, lifetime and impacts of newly-formed aerosol particles. This effect is expected to be particularly important for unpolluted regions which are sensitive to this secondary aerosol source^48^. This includes preindustrial conditions, which are an important source of uncertainty in the overall estimates on anthropogenic effects on clouds and climate^6,49^.

It can be noted that theoretical approaches other than the standard GDE-based-methods, such as Monte Carlo simulations^50^, can be applied to avoid the issues related to the continuum approximation. However, the GDE and especially the straight-forward continuum condensation rate calculations will undoubtedly remain a central tool to analyze measurements. The entire particle size range can be addressed by discrete-sectional GDE models, which include also coagulation and other dynamic processes. Furthermore, while the simulations of this work are in terms of measurable, dimensional quantities, GDE models can also be made non-dimensional for efficient probing of the parameter space^51^. The discrete-sectional models apply the discrete GDE for the smallest sizes and the continuous GDE for larger particles, once more highlighting the need to locate the size regime starting from which the continuous description is applicable.

We show that the onset of continuous condensational growth can be assessed based on an observed particle size distribution by using the ratio between the second- and first-order derivatives of the size distribution function as a metric. While the presented case studies address airborne nanoparticle formation, the rationale behind the metric applies to any physical and chemical systems involving particle formation and growth. The proposed tool gives direct information on the sizes at which the transition from discrete to continuous modeling can be done with reasonable accuracy, which (1) ensures correct interpretation of observations, and (2) enables reliable assessment of parameters controlling the particle formation process from experimental data.

## Methods

### Simulations based on the discrete GDE

The time evolution of the nanoparticle concentrations was simulated by solving the complete discrete GDE as given by Eq. (1), including collisions with and evaporations of vapor molecules, coagulation among the particles, and a sink reducing the vapor and particle concentrations. The collision rate constants *β*_*i*,*j*_ were calculated as hard-sphere collision rates, and the evaporation rate constants *γ*_*i*+*j* → *i*,*j*_ were obtained as described in Supplementary Information Section 1. Particle fission was omitted. Details of the simulated compounds and simulation set-ups, and of the numerical solution method are found in Supplementary Information Section 1. To avoid unnecessary computational burden, the size distribution was truncated at ca. 5–10 nm depending on the chemical system, ensuring that the truncation size was beyond the sizes at which non-stochastic condensation begins to dominate.

### GR_app_ based on the appearance times of different particle sizes

To analyze the data similarly to experiments, the apparent growth rate GR_app_ (Eq. (7)) was determined by applying linear fits on the (*t*_app_, *d*_p_)-curve^38,39,45^. For each size bin, the fit included five adjacent data points centered at the bin. However, GR_app_ is not sensitive to the exact number of points included: including three points or simply taking the numerical derivative give similar, but slightly more scattered results.

### GR_cond_ calculated from molecular collision and evaporation rates

The continuous condensational growth rate GR_cond_ was calculated according to Eq. (6), including in the mass flux also very small clusters in case that they were present at relatively high concentrations. The reason for this is that the concentrations of the smallest clusters consisting of only a couple of molecules may become non-negligible, and omitting them in the growth rates of larger particles leads to small underestimation of GR_cond_ (Fig. 1b). For size-binned data, GR_cond_ was determined by representing each bin with the particle size at the bin midpoint. For the LVOC–ELVOC mixture, the representative composition of a size bin was calculated as the weighted average over the compositions of all individual particles belonging to the bin.

The following approach was used to determine which small clusters are included in GR_cond_ for each size bin: The collision frequencies *β*_bin_*C*_bin_ of smaller bins with the given bin were compared to the collision frequency ∑*β*_vapor_*C*_vapor_ of vapor molecules with the bin. The relative contribution of different smaller bins depends on the bin width; on the other hand, the particle concentration typically decreases as a function of size with the smallest sizes having clearly the highest concentrations. Therefore, all smaller size bins up to *n*_bin,max_ were included in GR_cond_ if the total collision frequency $\,{\sum }_{1}^{{n}_{{\rm{bin}},{\rm{\max }}}}{\beta }_{{\rm{bin}}}{C}_{{\rm{bin}}}$ was at least 0.01 times the condensation frequency ∑*β*_vapor_*C*_vapor_, and including more bins in the sum had no further effect. It must be noted that this approach is applicable only if the coagulational growth involves solely the clustering of very small, vapor-like molecular clusters onto considerably larger particles. If self-coagulation among the studied particle size range is significant, the apparent growth cannot be described solely by mass flux calculations (Eq. (6)), but instead the full GDE must be applied. Therefore, simulation conditions that led to self-coagulation were excluded from the comparisons of GR_app_ and GR_cond_, and were analyzed by TREND instead.

### GR_TREND_ determined by the full-GDE-based analysis tool TREND

The details of the TREND method are found in ref.^31^. Briefly, the modeled distribution is calculated within TREND using as a starting point the measured (in this case synthetic) distribution at an earlier point in time. TREND solves the GDE for a given time interval and size resolution considering all quantifiable mechanisms that alter the aerosol size distribution, including coagulation and particle sinks. As a result, particle growth of any form, including both deterministic and stochastic contributions, remains the only unknown, and is determined by comparing fractions of the modeled and measured particle size distributions after the modeled time interval. The procedure starts at the largest particles of the modeled distribution and assigns size intervals containing a constant number of particles. This is repeated for the measured distribution and the corresponding size intervals containing the same number of particles are identified. Relating the count medium diameter of both intervals to each other allows assessing the growth rate, which may also be negative in case of particle shrinkage.

The analysis tool was adopted to the specifications of the synthetic molecular-resolution data. First, the toolkit was modified to accept the mass and number concentration data from the molecular-resolution model, converting them to size-binned concentration using a bin width of 2% of the corresponding lower bin limit. Second, only the largest 1%, or 10% in case of higher vapors source rates (4–5)∙10^4^ cm^−3^s^−1^, of the particle size distribution were analyzed with the method. This is in order to avoid significantly limiting the size resolution of the TREND method, as the vast majority of the particles are contained within the first molecular clusters. However, note that all clusters except for the monomers are considered for simulating the aerosol dynamics, i.e. coagulation of the smallest molecular clusters is taken into account. The obtained growth rates GR_TREND_ are thus compared to GR_cond,vapor_ calculated considering only vapor monomers. It must be noted that as TREND considers size intervals instead of discrete molecule-by-molecule sizes, some differences in the description of coagulation compared to the accurate discrete model can be expected.

### Determination of *∂*^2^:*∂*

The gradients of the simulated discrete distribution can be straight-forwardly determined as numerical derivatives. In practice, molecular-resolution observations are not at present possible for arbitrary compounds, and instruments that are used to measure size-dependent particle concentrations classify the particles into size bins according to the mobility diameter. In addition, multi-compound systems may exhibit more than one parallel particle growth pathways, and thus following the growth molecule-by-molecule is not unambiguous even if molecular-resolution observations are available.

The metric *∂*^2^:*∂* (Eq. (8)) was thus determined for size-binned particle distributions *c*′(*d*_p_) by fitting a suitable function to the distribution. The reliability of this approach was tested by applying the fit to a molecular-resolution distribution, and comparing the obtained ratio *∂*^2^:*∂* of the second and first analytical derivatives of the fitted distribution to that determined from the numerical derivatives of the discrete distribution, ensuring that the fit is able to reproduce the gradients.

The evaluation of the fitting approach for *∂*^2^:*∂* was conducted as described below.

1. The numerical derivatives of *c* (in cm^−3^ molec.^−1^) at each discrete cluster size *i*_0_ (molec.) were determined according to standard numerical differentiation approaches as
  9 $${\frac{\partial c}{\partial i}|}_{i={i}_{0}}=\frac{c({i}_{0}+{\rm{\Delta }}i)-c({i}_{0}-{\rm{\Delta }}i)}{2{\rm{\Delta }}i}$$
  and
  10 $${\frac{{\partial }^{2}c}{\partial {i}^{2}}|}_{i={i}_{0}}=\frac{c({i}_{0}+{\rm{\Delta }}i)-2c({i}_{0})+c({i}_{0}-{\rm{\Delta }}i)}{{({\rm{\Delta }}i)}^{2}},$$
  where Δ*i* = one molecule.
2. The number density *c*(*i*) with respect to the molecular content of the particles was converted to the number density *c*′(*d*_p_) with respect to particle diameter as *c*′ = *c* × d*i*/d*d*_p_. A third-order polynomial function was fit to the base-10 logarithm of *c*′ around the size of interest as demonstrated in Supplementary Fig. S2a, and ∂*c*′/∂*d*_p_ and ∂^2^*c*′/∂*d*_p_^2^ were obtained as analytical derivatives of the fit. The fit was applied piecewise around each particle size or size bin, as finding a function that is capable of reproducing the shape of a wider size range does not seem possible. For the limited size ranges, a 3^rd^ order polynomial function is able to capture typical trends in the concentration density, including monotonously decreasing or increasing behavior, decrease or increase with a plateau, and local minima and maxima.
3. The derivatives of the fit *c*′(*d*_p_) give the changes in the number density and its slope per unit diameter. In order to assess the gradients of the distribution with respect to molecular additions, corresponding to Eqs (2–4), the ratio *∂*^2^:*∂* of the derivatives of *c* with respect to *i* was obtained from the derivatives of *c*′ with respect to *d*_p_ as

11 $$\begin{array}{lll}{\partial }^{2}:\partial  & = & \frac{\frac{{\partial }^{2}c}{\partial {i}^{2}}}{\frac{\partial c}{\partial i}}=\frac{\frac{{\rm{d}}{d}_{{\rm{p}}}}{{\rm{d}}i}\frac{\partial }{\partial {d}_{{\rm{p}}}}[\frac{{\rm{d}}{d}_{{\rm{p}}}}{{\rm{d}}i}\frac{\partial }{\partial {d}_{{\rm{p}}}}(c^{\prime} \frac{{\rm{d}}{d}_{{\rm{p}}}}{{\rm{d}}i})]}{\frac{{\rm{d}}{d}_{{\rm{p}}}}{{\rm{d}}i}\frac{\partial }{\partial {d}_{{\rm{p}}}}(c\text{'}\frac{{\rm{d}}{d}_{{\rm{p}}}}{{\rm{d}}i})}\\  & = & \frac{{\rm{d}}{d}_{{\rm{p}}}}{{\rm{d}}i}\frac{\frac{{{\rm{d}}}^{2}}{{\rm{d}}{{d}_{{\rm{p}}}}^{2}}(\frac{{\rm{d}}{d}_{{\rm{p}}}}{{\rm{d}}i})c^{\prime} +2\frac{{\rm{d}}}{{\rm{d}}{d}_{{\rm{p}}}}(\frac{{\rm{d}}{d}_{{\rm{p}}}}{{\rm{d}}i})\frac{\partial c^{\prime} }{\partial {d}_{{\rm{p}}}}+\frac{{\rm{d}}{d}_{{\rm{p}}}}{{\rm{d}}i}\frac{{\partial }^{2}c^{\prime} }{\partial {{d}_{{\rm{p}}}}^{2}}}{\frac{{\rm{d}}}{{\rm{d}}{d}_{{\rm{p}}}}(\frac{{\rm{d}}{d}_{{\rm{p}}}}{{\rm{d}}i})c^{\prime} +\frac{{\rm{d}}{d}_{{\rm{p}}}}{{\rm{d}}i}\frac{\partial c^{\prime} }{\partial {d}_{{\rm{p}}}}}+\frac{{\rm{d}}}{{\rm{d}}{d}_{{\rm{p}}}}(\frac{{\rm{d}}{d}_{{\rm{p}}}}{{\rm{d}}i}).\end{array}$$

In Eq. (11), d*d*_p_/d*i* and its derivatives with respect to *d*_p_ are calculated from the molecular volume assuming spherical particles.

Supplementary Fig. S2b shows that the fit-based *∂*^2^:*∂* reproduces the numerical-derivative-based results very well, indicating that the shape of the distribution can be reliably captured by the fit. In addition, the hypothesis that the relative importance of the drift and diffusion terms ($-\,\frac{\partial }{\partial i}[(\beta {C}_{1}-\gamma )c]$ and $\frac{1}{2}\frac{{\partial }^{2}}{\partial {i}^{2}}[(\beta {C}_{1}+\gamma )c]$, respectively) in Eq. (3) is reflected in the derivatives of the distribution ∂*c*/∂*i* and ∂^2^*c*/∂*i*^2^ was verified by comparing the ratio of the terms to the ratio of the derivatives, i.e. *∂*^2^:*∂*, for representative simulation cases, as discussed in Supplementary Information Section 1.4.

### Effect of size resolution on *∂*^2^:*∂*

Sub-3 nm particle concentrations are often measured with diethylene-glycol-based particle counters, such as Particle Size Magnifier (PSM)^13^. We have thus chosen to by default bin the simulation data according to the best size resolution reported for PSM, namely 0.1 nm^45^, and tested the sensitivity of *∂*^2^:*∂* to the size resolution by using different bin widths between 0.2 and 1.0 nm. Supplementary Fig. S4a demonstrates the fitting approach applied on size-binned data. As the bins may contain different numbers of particles, the binned distribution is less smooth especially towards the smallest sizes. However, the shape of the distribution can still be represented by the fit, as shown in Supplementary Fig. S4b. *∂*^2^:*∂* obtained with bin widths of Δ*d*_p_ = 0.5 and 1.0 nm differ more from the accurate result, but reproduce the correct trend, order of magnitude, and size around which *∂*^2^:*∂* decreases to a few percent. It must be noted that for smaller molecules, a given bin Δ*d*_p_ contains more discrete particle compositions, and the resolution in terms of the molecular content becomes lower. However, for the largest bin widths studied, the bins contain up to tens or even hundreds of particle sizes, demonstrating that the overall behavior of *∂*^2^:*∂* is not distorted by an imperfect size resolution.

## Electronic supplementary material

Supplementary information

## Data Availability

The datasets generated and analysed during the current study are available from the corresponding author on reasonable request.
