# Supplementary material for: Robust metric for quantifying the importance of stochastic effects on nanoparticle growth
Source: Sci Rep. 2018 Sep 21;8:14160. doi: 10.1038/s41598-018-32610-z (PMC6154961; doi:10.1038/s41598-018-32610-z)
Supplement: Supplementary file 1 — Supplementary information [file 41598_2018_32610_MOESM1_ESM.pdf]

Supplementary information for:

Robust metric for quantifying the importance of stochastic effects on nanoparticle growth

Tinja Olenius, Lukas Pichelstorfer, Dominik Stolzenburg, Paul M. Winkler, Kari E. J. Lehtinen and Ilona Riipinen

1 Supplementary Methods

1.1 Simulated molecules and particles

The simulated molecules are representative of atmospheric low-volatile and extremely-low-volatile organic compounds (LVOC and ELVOC, respectively). The properties of these species, listed in Supplementary Table S 1, are based on ref. 1, and result in apparent growth rates GR_{app} similar to those observed. We simulate both a one-compound LVOC system and a two-compound LVOC–ELVOC system. The simulated particle size range includes all discrete particle compositions from single vapor molecules to particles of a mobility diameter of d_p = 10 nm in case of LVOC, and d_p = 4.5 nm in case of LVOC–ELVOC mixture. The size range is smaller for the two-compound system, as the number of all possible individual molecular compositions in a given size range, and consequently the number of coupled differential equations in the discrete GDE (Eq. (1)), increases drastically for systems of multiple chemical compounds. For LVOC–ELVOC, the size range up to 4.5 nm contains already 3730 individual particle compositions, and including larger sizes rapidly becomes computationally unaffordable. For LVOC, the size range up to 10 nm contains 1350 discrete particle sizes. In order to be able to study wider size ranges, we focus on the LVOC system, but report results also for the LVOC–ELVOC mixture to demonstrate that the presented analysis tools are valid also for multi-compound systems. The saturation vapor pressure of 10^{-10} Pa used for ELVOC is somewhat higher than the lowest reported estimates of ca. 10^{-13}–10^{-12} Pa[1]. The higher value was used as (1) it has been proposed that these types of compounds may not have as low saturation vapor pressures as often assumed[2], and (2) using lower values in the simulations results in very high particle concentrations and strong particle self-coagulation, making condensational growth less important in the sub-5 nm size range.

The evaporation rate of compound k from particle of size d_p is in most simulations approximated with the Kelvin formula

$$\gamma_k = \beta_k \frac{p_{\mathrm{sat},k}}{k_B T} \chi_k \exp\left(\frac{4\sigma m_k}{k_B T \rho_p d_p}\right), \quad \text{(S1)}$$

where β_k is the rate constant of the corresponding molecular collision, and $p_{\mathrm{sat},k}$ and χ_k are the pure liquid saturation vapor pressure and the particle-phase mole fraction of k, respectively. σ is the particle surface tension, k_B is the Boltzmann constant, and T is the temperature. In addition, we perform simulations using qualitatively and quantitatively different evaporation rates to show that the obtained conclusions are independent of these rates.

The default simulation set-up corresponds to a laboratory chamber experiment (Supplementary Table S 2). The initial concentrations of vapors and particles are set to zero, a constant vapor source is turned on, and the simulation is run until the concentrations of particles in the simulated size range have reached a

steady state. The walls of the chamber and the dilution of the chamber air act as sinks for the vapors and particles (Supplementary Table S 2).

Supplementary Table S 1: Properties of the simulated representative organic vapors and particles.

**Property**	**Species**		
	**LVOC**	**ELVOC**	**Particles**
Mass (amu)	300	500	–
Saturation vapor pressure (Pa)	10^{-9}	10^{-10}	–
Density (kg m^{-3})	1400	1400	1400
Surface tension (N m^{-1})	–	–	$2.3 \cdot 10^{-2}$

Supplementary Table S 2: Parameters related to the simulation conditions.

<table>
<tr><th>Parameter</th><th colspan="2">Value and/or functional form</th></tr>
<tr><td>Temperature T</td><td colspan="2">278 K</td></tr>
<tr><td rowspan="2">Vapor source Q ($cm^{-3}s^{-1}$) and corresponding final vapor concentration C (cm^{-3})</td><td>Simulations of LVOC</td><td>$Q = 8 \cdot 10^3 - 6 \cdot 10^4$ $cm^{-3}s^{-1}$
$C = 8 \cdot 10^6 - 6 \cdot 10^7$ cm^{-3}</td></tr>
<tr><td>Simulations of LVOC–ELVOC mixture</td><td>Total vapor source rate $Q = 8 \cdot 10^3 - 5 \cdot 10^4$ $cm^{-3}s^{-1}$, ELVOC:LVOC ratio 10:90, test simulations with a 50:50 ratio</td></tr>
<tr><td rowspan="2">Vapor and particle sink rate constant S (s^{-1})</td><td>Sink corresponding to the walls of the laboratory chamber CLOUD</td><td>$S(d_p) = 10^{-3}$ nm s^{-1} / $d_p + 9.6 \cdot 10^{-5}$ s^{-1},
where d_p is the mobility diameter of a particle or vapor molecule, and the size-independent term corresponds to dilution of the chamber air[17]</td></tr>
<tr><td>Sink corresponding to the surface of larger aerosol particles in the ambient atmosphere</td><td>$S(d_p) = S_{ref} \cdot (d_p / d_{p,ref})^{-1.6}$,
where S_{ref} and $d_{p,ref}$ are a reference sink and size corresponding to LVOC vapor[15]. S_{ref} was set to either 10^{-3} or $5 \cdot 10^{-4}$ s^{-1}.</td></tr>
</table>

1.2 Additional simulation set-ups

To examine the effect of external conditions on the apparent growth of the particle population, additional simulations were performed using a different size dependence and magnitude for the sink rate, as well as using no sinks and a time-independent vapor concentration. The alternative sink rate (Supplementary Table S 2) corresponds to scavenging by a background population of larger aerosol particles. In addition, test simulations were performed using the default chamber set-up but different properties for the vapor and particles. The mass, density, surface tension and saturation vapor pressure were set to $m = 98.08$ amu, $\rho = 1830$ kg m^{-3}, $\sigma = 5\cdot10^{-2}$ N m^{-1}, and $p_{sat} = 10^{-9}$ Pa, respectively, corresponding to quasi-unary, stabilized sulfuric acid[3].

Finally, test simulations were conducted using different particle evaporation rate profiles. While accurate assessments of evaporation rates of small clusters remain unavailable, the rates are expected to exhibit discrete changes as a function of cluster size and composition instead of a smooth behavior such as given by Eq. (S1)[4,5]. This affects the details of the particle distribution, and thus we performed test simulations using different types of non-smooth evaporation profiles. These rate profiles correspond to the qualitative behavior and order of magnitude of evaporation rates calculated with different quantum chemical methods[4–8]. The rate profiles were obtained as follows: Rates calculated by the Kelvin approach using the LVOC properties were taken as a starting point, and random noise was introduced to the rates, with the magnitude of the noise decreasing as a function of particle size as

$$\gamma_{\mathrm{mod}} = 10^{\left[\Delta OoM\left(-1+2R\{0\ldots1\}\right)\exp\left(1-N^{0.2}\right)\right]}\gamma_{\mathrm{Kelvin}}, \quad \text{(S2)}$$

In Eq. (S2), γ_{mod} and γ_{Kelvin} are the modified and the Kelvin-formula-based evaporation rate constants, respectively, ΔOoM is the maximum change in the order of magnitude of the rate, and $R\{0\ldots1\}$ is a randomly generated number between 0 and 1. The purpose of the exponential factor is to decrease the absolute modification as the number of molecules N in the particle increases, making γ_{mod} approach the macroscopic rate γ_{Kelvin}. Two sets of modified rates were generated using maximum changes of $\Delta OoM = 2$ and $\Delta OoM = 3$. These tests do not address the chemical accuracy of the properties assigned to the compounds; instead, the aim is to use evaporation rates of a realistic order, and study the effect of the shape of the rate profile on the applicability of the presented analysis tools.

In addition, test simulations were performed for representative sulfuric acid–ammonia (H_2SO_4–NH_3) and sulfuric acid–dimethylamine (H_2SO_4–DMA) systems using available quantum chemical data for the smallest clusters (Supplementary Table S 3). The data were taken from ref. 6 for H_2SO_4–NH_3, and from ref. 7 for H_2SO_4–DMA. For evaporation rates of larger particles, we included two different scenarios (Supplementary Table S 3): (1) The rates were computed from the standard Kelvin formula (Eq. (S1)) with p_{sat} set to result in realistic vapor and particle concentrations. (2) The rates were set to include a prefactor Γ, corresponding to an activity coefficient for non-ideal solutions, in order to mimic the strong acid–base interaction originating from hydrogen bonding and salt formation in the particle phase.

Examples of the resulting particle distributions for the different simulated systems are shown in Supplementary Figure S 1. All simulation results are in line with the conclusions. Representative results for the metric $\partial^2{:}\partial$ are included in Figure 2b, and the different molecular mixtures are discussed in Supplementary Section 2.1.

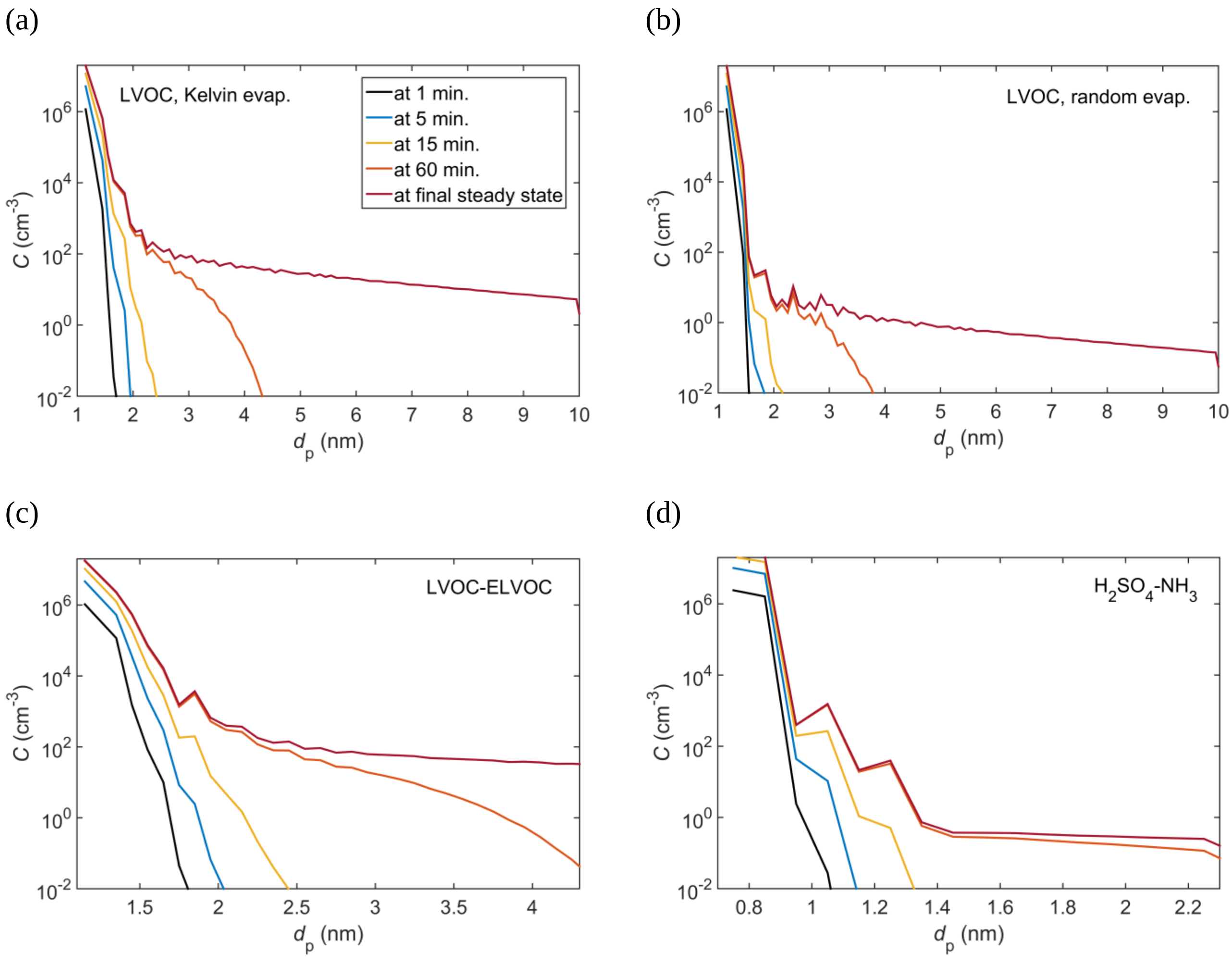

Supplementary Figure S 1: Examples of the size-dependent particle concentration at different moments in time for different simulation systems. Panel (a): LVOC with the evaporation rates from Eq. (S1) at final vapor concentration of C_{LVOC} = $2\cdot10^{7}$ cm^{-3}. Panel (b): LVOC with the evaporation rates from Eq (S2) (ΔOoM = 2) at $C_{\mathrm{LVOC}} = 2\cdot10^{7}$ cm^{-3}. Panel (c): LVOC–ELVOC at $C_{\mathrm{LVOC}} = 2\cdot10^{7}$ cm^{-3} and $C_{\mathrm{ELVOC}} = 2\cdot10^{6}$ cm^{-3}. Panel (d): H_2SO_4–NH_3 at $C_{\mathrm{H2SO4}} = 2\cdot10^{7}$ cm^{-3} and $C_{\mathrm{NH3}} = 3\cdot10^{7}$ cm^{-3}.

Supplementary Table S 3: Test simulations including quantum-chemistry-based (QC) evaporation rates for the smallest clusters.

Simulation case with QC data		Details of the simulation rate constants
H_2SO_4–NH_3	1	Evaporation rates γ for clusters consisting of up to 5 H_2SO_4 and 5 NH_3 molecules from QC[6,18], and for larger particles from Eq. (S1) with $p_{sat,H_2SO_4} = 10^{-9}$ Pa, $p_{sat,NH_3} = 10^{-8}$ Pa, $\rho_{H_2SO_4}$ = 1830 kg m^{-3}, ρ_{NH_3} = 696 kg m^{-3}, and $\sigma = 5 \cdot 10^{-2}$ N m^{-1}
	2	Otherwise same as H_2SO_4–NH_3 case 1, but with $p_{sat,H_2SO_4} = 10^{-8}$ Pa, $p_{sat,NH_3} = 10^{-7}$ Pa, and γ from Eq. (S1) modified to include an activity coefficient $\Gamma = \exp\,[5 \cdot (1 - \chi_k)]$, where χ_k is the mole fraction of the evaporating compound
	3	Quasi-unary H_2SO_4–NH_3 system with the molecular properties of H_2SO_4, and γ for the smallest clusters from QC along the main cluster growth pathway[18] and for larger particles from Eq. (S1) with m = 98.08 amu, $p_{sat} = 10^{-9}$ Pa, ρ = 1830 kg m^{-3}, and $\sigma = 5 \cdot 10^{-2}$ N m^{-1}
	4	Otherwise same as H_2SO_4–NH_3 case 3, but with all collision rate constants β enhanced by a factor of 2.3 to test possible electrostatic effects due to dipoles[19]
H_2SO_4–dime-thylamine (DMA)	1	Evaporation rates γ for clusters consisting of up to 4 H_2SO_4 and 4 DMA molecules from QC[7], and for larger particles from Eq. (S1) with $p_{sat,H_2SO_4} = 10^{-9}$ Pa, $p_{sat,DMA} = 10^{-9}$ Pa, $\rho_{H_2SO_4}$ = 1830 kg m^{-3}, ρ_{DMA} = 680 kg m^{-3}, and $\sigma = 5 \cdot 10^{-2}$ N m^{-1}
	2	Otherwise same as H_2SO_4–DMA case 1, but with $p_{sat,H_2SO_4} = 10^{-8}$ Pa, $p_{sat,DMA} = 10^{-8}$ Pa, and γ from Eq. (S1) modified to include an activity coefficient $\Gamma = \exp\,[5 \cdot (1 - \chi_k)]$, where χ_k is the mole fraction of the evaporating compound

1.3 Numerical solution of the discrete GDE

The discrete GDE (Eq. (1)) was solved by a standard Euler method using an adaptive time step. The step size is adjusted as follows: At every integration step, the initial estimate for the step size Δt is determined based on the previous integration step and the maximum allowed relative change $\delta C_{\max} = 0.01$ in the concentration of an individual particle i. The former, referred to as Δt_{new}, is given by Eq. (S4) below, and the latter, referred to as Δt_{max}, is obtained from the initial concentrations $C_i\,(t_0)$ and their derivatives $\mathrm{d}C_i\,/\,\mathrm{d}t$ as

$$\Delta t_{\max} = \delta C_{\max} \times \min_i \left(\frac{C_i(t_0)}{\left| \frac{\mathrm{d}C_i}{\mathrm{d}t} \right|_{t=t_0} \right|} \right). \tag{S3}$$

The time step Δt is set to be the smaller one of Δt_{new} and Δt_{max}, and particle concentrations after Δt are evaluated using a single integration step. The time interval Δt is then integrated again using two steps of length Δt / 2, re-evaluating the derivatives $\mathrm{d}C_i$ / $\mathrm{d}t$ at the midpoint. The two solutions for each i are compared, and if their relative difference ε does not exceed a set tolerance of $\varepsilon_{\max} = 10^{-5}$, Δt is accepted. Otherwise, Δt is decreased as[9]

$$\Delta t_{\text{new}} = 0.8 \left(\frac{\varepsilon_{\max}}{\max_i(\varepsilon)} \right)^{1/2} \Delta t, \tag{S4}$$

and a new attempt is made until the criterion regarding $\varepsilon_{\max}$ is satisfied. After successful integration, an estimate Δt_{new} for the next time step is determined according to Eq. (S4), and the procedure continues as described above. The criteria $\varepsilon_{\max}$ and $\delta C_{\max}$ apply to all particles that have a concentration of at least 10^{-12} cm^{-3}. The Eulerian integration was tested against the Fortran ODE solver VODE[10] using small simulation systems, ensuring that the method is reliable.

When a collision leads to a particle outside of the simulated size range, the particle is placed in an additional size bin, which represents all larger particles grown out of the simulation range. Particles in the additional bin have the same size corresponding to the size of particles at the boundary of the simulation range. These outgrown particles, the purpose of which is to account for the additional coagulational loss caused by larger particles, can coagulate with the simulated particles and each other and be lost to the sink, but they do not evaporate or grow in size.

1.4 Evaluation of the methods for determining $\partial^2{:}\partial$

Supplementary Figure S 2, Supplementary Figure S 3 and Supplementary Figure S 4 demonstrate the evaluation of the proposed metric $\partial^2{:}\partial$, as discussed in Methods. As demonstrated in Supplementary Figure S 3, the ratio of the derivatives of the concentration c (i.e. the metric $\partial^2{:}\partial$) indeed exhibits a similar size-dependent behavior as that of the terms in Eq. (3). The singular point in the ratio of the terms (green solid line) is due to the drift flux $(\beta C_1 - \gamma)c$ exhibiting a turning point where its derivative is zero. The connection between the derivatives of the concentrations and those of the fluxes can be further elucidated by writing out the terms in Eq. (3):

$$\underbrace{-\frac{\partial}{\partial i}[(\beta C_1 - \gamma)c]}_{\text{cont.}} + \frac{1}{2}\frac{\partial^2}{\partial i^2}[(\beta C_1 + \gamma)c] = \beta C_1 \left(\underbrace{-\frac{\partial c}{\partial i}}_{\text{cont.}} + \frac{1}{2}\frac{\partial^2 c}{\partial i^2} \right) + \gamma \left(\underbrace{\frac{\partial c}{\partial i}}_{\text{cont.}} + \frac{1}{2}\frac{\partial^2 c}{\partial i^2} \right) + \frac{\partial(\beta C_1)}{\partial i}\left(\underbrace{-c}_{\text{cont.}} + \frac{\partial c}{\partial i} \right) + \frac{\partial \gamma}{\partial i}\left(\underbrace{c}_{\text{cont.}} + \frac{\partial c}{\partial i} \right) + \frac{1}{2} c \frac{\partial^2}{\partial i^2}(\beta C_1 + \gamma). \tag{S5}$$

In Eq. (S5), the terms of the continuous condensation equation (Eq. (4)) are marked with "cont.", and the rest of the terms must be negligible for the continuous assumption to hold. The first terms on the right-hand side of Eq. (S5) demonstrate that $\partial^2 c/\partial i^2$ needs to be minor compared to $\partial c/\partial i$ in order to describe vapor condensation and evaporation neglecting stochastic widening, also when other dynamic processes affect the size distribution.

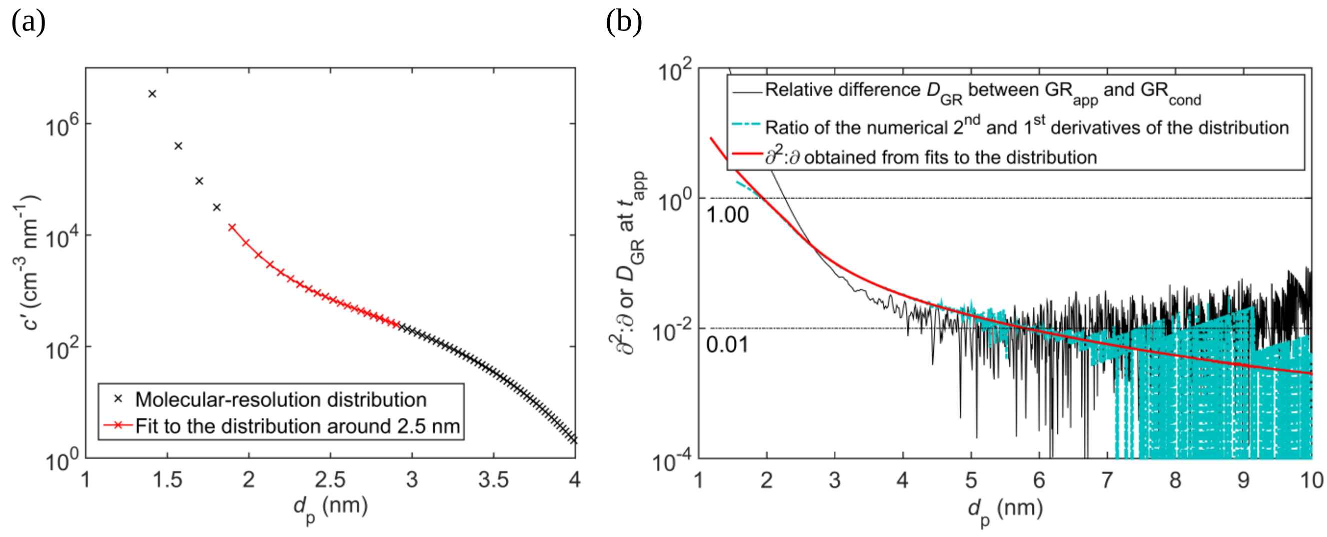

Supplementary Figure S 2: Panel (a): Particle number density $c'(d_p)$ of a molecular-resolution distribution for LVOC at $C_{LVOC} = 2 \cdot 10^7$ cm^{-3}, and a 3rd order polynomial fit to the distribution around $d_p = 2.5$ nm. Panel (b): Ratio of the numerical derivatives of $c(i)$ (Eqs. (9) and (10); dash-dotted turquoise line), and $\partial^2{:}\partial$ obtained from the fit to $c'(d_p)$ and Eq. (11) (solid red line).

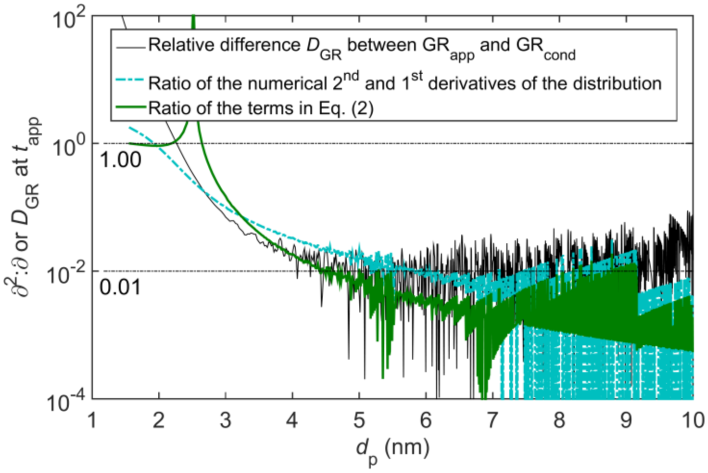

Supplementary Figure S 3: Ratio of the numerical derivatives of $c(i)$ (Eqs. (9) and (10); dash-dotted turquoise line), and ratio of the diffusion and drift terms in Eq. (3) (solid green line), determined numerically in the same way as the derivatives of $c(i)$.

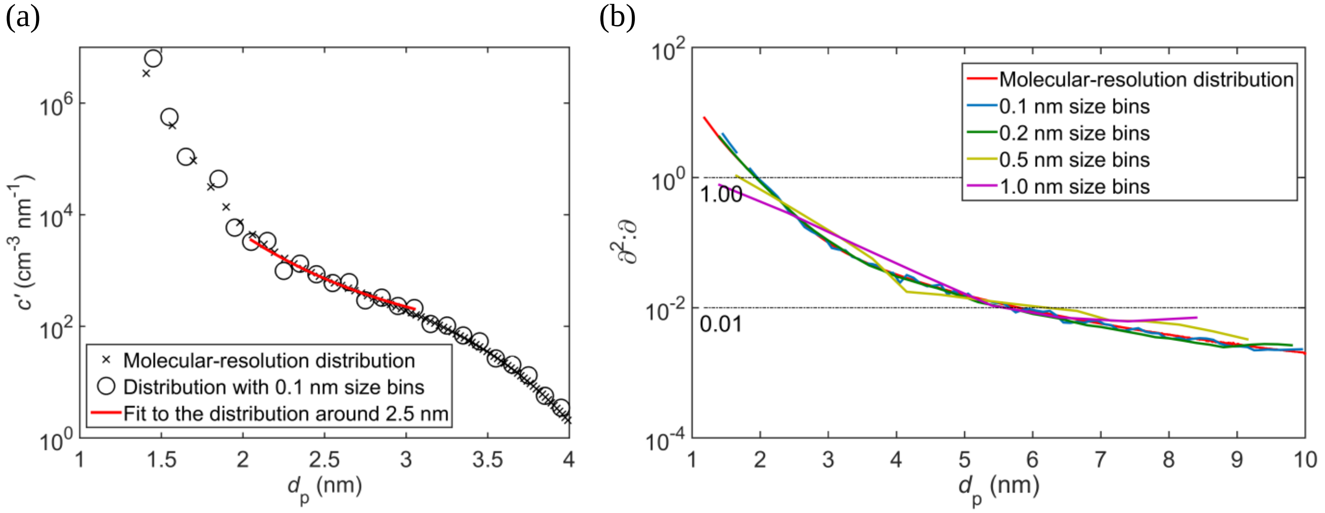

Supplementary Figure S 4: Panel (a): Particle number density $c'(d_p)$ assuming a size resolution of 0.1 nm for LVOC at $C_{LVOC} = 2{\cdot}10^7$ cm^{-3} (black circles), and a 3rd order polynomial fit to the size-binned distribution around $d_p = 2.5$ nm (solid red line). The molecular-resolution distribution (Supplementary Figure S 2a) is shown for reference (black crosses). Panel (b): $\partial^2{:}\partial$ from fits to the size-binned distribution for different size resolutions.

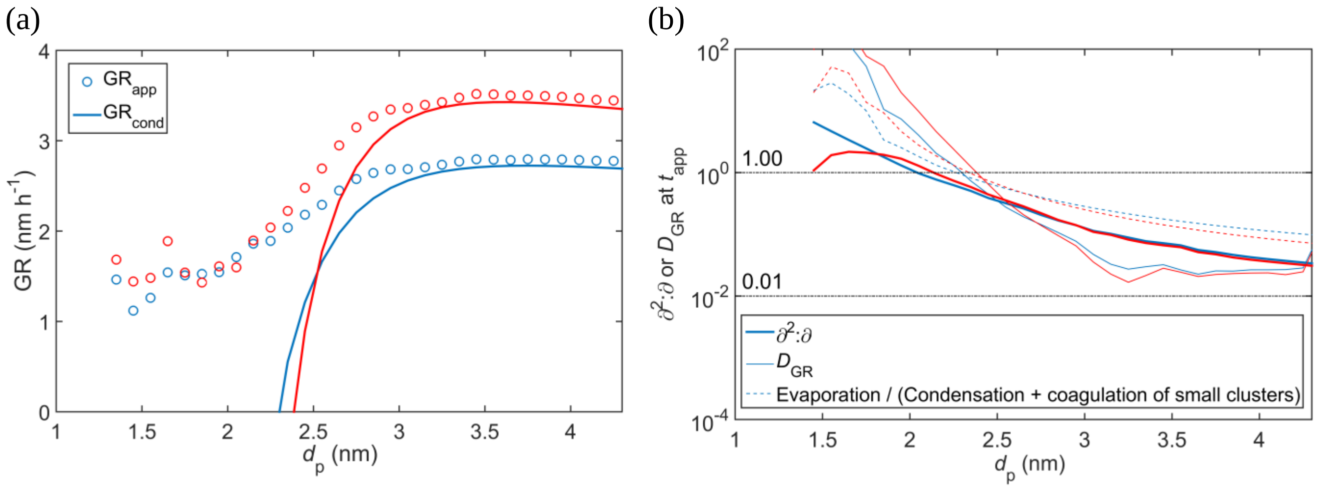

Supplementary Figure S 5: Panel (a): GR_{app} and GR_{cond} for the LVOC–ELVOC mixture at chamber conditions at a total final vapor concentration of $C_{vapor} = 2{\cdot}10^7$ cm^{-3}, with LVOC:ELVOC ratios of 10:90 (blue) and 50:50 (red). Panel (b): $\partial^2{:}\partial$ and the relative difference D_{GR} between GR_{app} and GR_{cond}.

2 Supplementary Discussion

2.1 Two-compound mixtures

The mixture of LVOC and ELVOC compounds was modeled assuming conditions similar to the LVOC simulations, but splitting the vapor source in a 90:10 ratio between LVOC and ELVOC according to the relative abundances of these types of compounds assessed by laboratory measurements[1]. A test simulation was performed using a 50:50 split. The behavior of GR_{app} and GR_{cond} is similar to the LVOC system

(Supplementary Figure S 5a), but the absolute values are increased by the lower-volatility compound. As for the LVOC simulations, the metric $\partial^2{:}\partial$ is able to predict the size range where GR_{app} and GR_{cond} converge (Supplementary Figure S 5b; Figure 2b). Also all the H_2SO_4–base systems simulated applying quantum chemical data (Supplementary Table S 3) behave similarly, examples of which are shown in Supplementary Figure S 6.

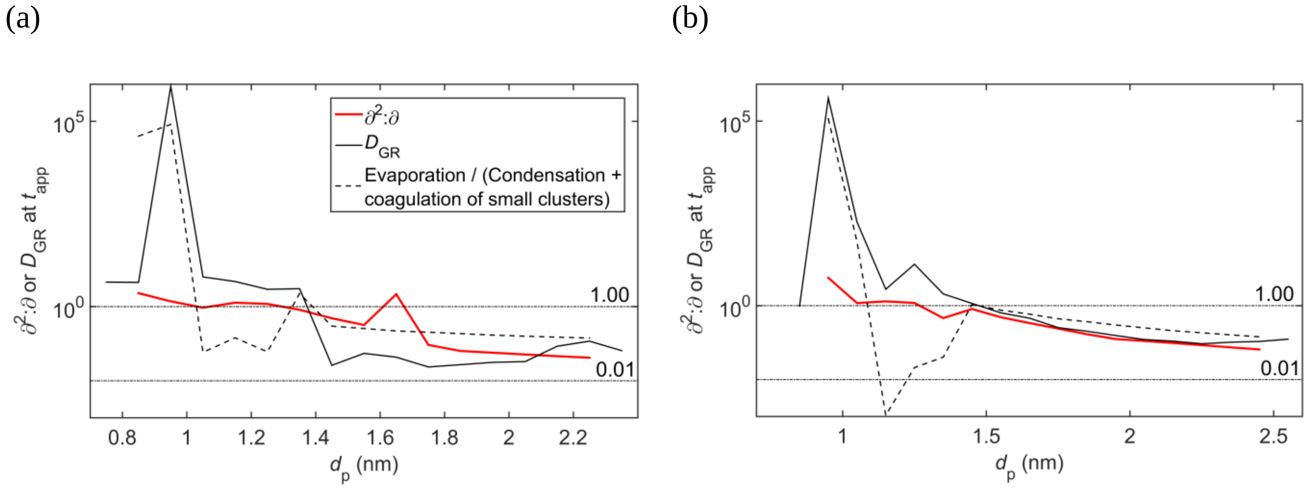

Supplementary Figure S 6: $\partial^2{:}\partial$ and the relative difference D_{GR} between GR_{app} and GR_{cond} for the H_2SO_4–base systems, scenario 1, at chamber conditions. Panel (a): H_2SO_4–NH_3 system at final vapor concentrations of $C_{H2SO4} = 2{\cdot}10^7$ cm^{-3} and $C_{NH3} = 3{\cdot}10^7$ cm^{-3}. Panel (b): H_2SO_4–DMA system at final vapor concentrations of $C_{H2SO4} = 10^7$ cm^{-3} and $C_{DMA} = 10^7$ cm^{-3}.

2.2 Comparison of time-dependent TREND results GR_{TREND} to $GR_{cond,vapor}$

Figure 1b, Figure 3 and Supplementary Figure S 7 present the comparison of GR_{TREND} determined with the TREND method to the pure condensational growth rate $GR_{cond,vapor}$ due to vapor monomers (Eq. (6)) for a set of different model cases. Obviously, the stochastic processes, which lead to increased GR_{app}, are quantified differently by TREND: at the beginning of the formation event, GR_{TREND} is high at the small sizes similar to GR_{app}, but the values decrease close to zero at later moments in time (Supplementary Figure S 7). This is because TREND gives an average growth rate based on the shift of specific subsections within the size distribution. At the beginning, the initial sizes build up, forming a "moving front", and TREND sees a positive shift towards larger sizes. After the initial build-up of the distribution, there exist larger particles that evaporate back towards the smallest sizes, and the forward and backward particle fluxes together result in a minor shift in TREND. In any case, it is important to note that GR_{TREND} is determined under the assumption that particles of a given size and at a given point in time grow at the same rate. As a result a particle that is larger than another particle is assumed to remain larger – no matter if there is growth or shrinkage. This is generally not the case in the size range governed by stochastic growth, and similarly to GR_{app}, GR_{TREND} cannot be interpreted through condensation at these sizes (see also Figure 2a). However, the benefit of TREND is that it excludes the effects of particle sinks and coagulation, and gives also the time dependence of the growth rates.

(a)

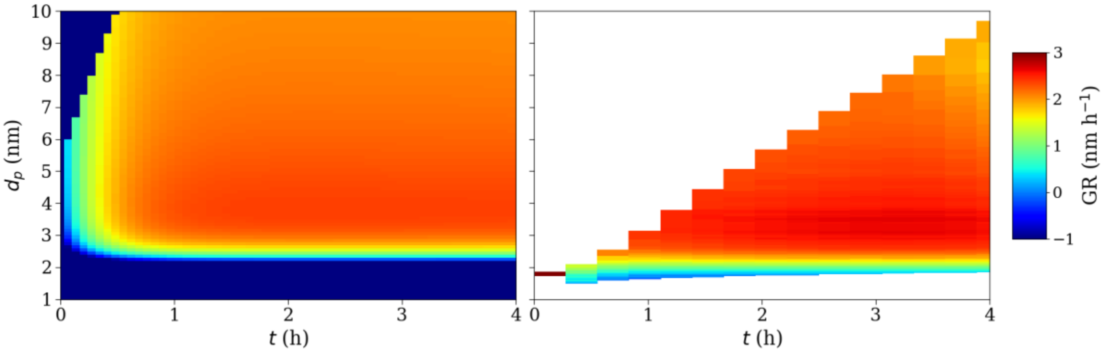

(b)

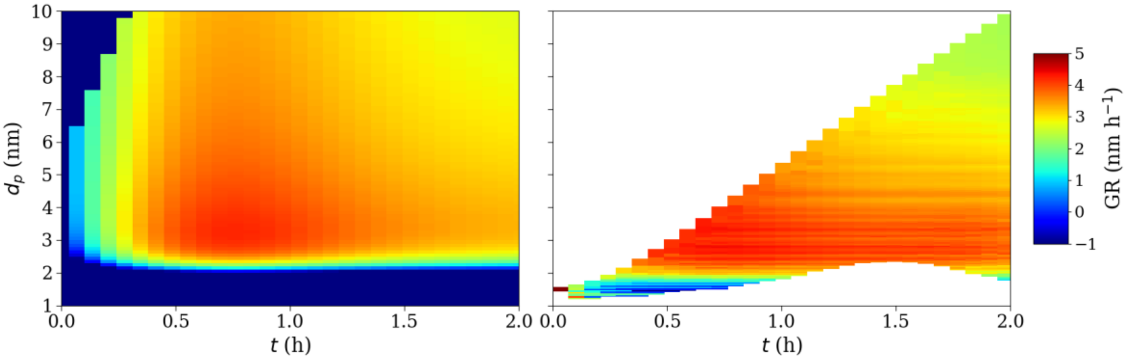

Supplementary Figure S 7: $GR_{cond,vapor}$ (left panels) and GR_{TREND} (right panels) as a function of particle size and time for LVOC at a vapor source rate of $Q = 2 \cdot 10^4$ cm^{-3} s^{-1} (panels (a)) and $4 \cdot 10^4$ cm^{-3} s^{-1} (panels (b)), which correspond to final vapor concentrations of $C_{LVOC} = 2 \cdot 10^7$ cm^{-3} and $3 \cdot 10^7$ cm^{-3}, respectively. Note that $GR_{cond,vapor}$ is calculated here considering only single vapor molecules to be consistent with GR_{TREND}.

Supplementary Figure S 7 shows the size- and time-dependent GR_{TREND} together with $GR_{cond,vapor}$ for representative LVOC simulations in which the growth is either driven by vapor and small clusters (panel (a)), or particle coagulation has a significant role (panel (b)). To compare the TREND results to those obtained using GR_{app}, Figure 3 presents GR_{TREND} at the particle appearance times for the LVOC and also for the LVOC–ELVOC system at different vapor source levels. For high vapor sources which generate strong particle formation and coagulation, GR_{TREND} and $GR_{cond,vapor}$ show stronger size- and time-dependence due to varying vapor concentration levels (Supplementary Figure S 7b), caused by the large number of formed particles becoming a significant sink for vapor. For these cases, GDE-based methods such as TREND are needed to extract the condensational growth rate, as GR_{app} cannot be interpreted solely through condensation even for the larger sizes (Figure 3).

2.3 Effect of the definition of t_{app} on GR_{app} vs. GR_{cond}

In this work, the appearance time t_{app} of a given particle size is by default defined according to the 50 % criterion, and GR_{cond} is accordingly calculated and compared to GR_{app} at this moment. This definition is however a matter of choice, and different criteria, including a 5 % criterion, have also been used[11]. Supplementary Figure S 8a shows the size- and time-dependent region where the continuous condensation

model is expected to be valid, based on ∂^2:∂ decreasing below five percent, together with t_{app} defined according to different criteria. The graph demonstrates that the choice of t_{app} affects the uncertainties related to the apparent growth rates: D_{GR} is larger for GR_{app} defined according to the 5 % criterion (Supplementary Figure S 8b). At the beginning of the formation event, the size-dependent particle concentration is steeper as the distribution at larger sizes has not yet built up, and the higher gradient affects the diffusion term in Eq. (3). Based on this, it is recommended that t_{app} is defined such that GR_{app} is not determined until the distribution around the size of interest has taken a less steep shape, for example according to the 50 % criterion instead of the 5 % criterion.

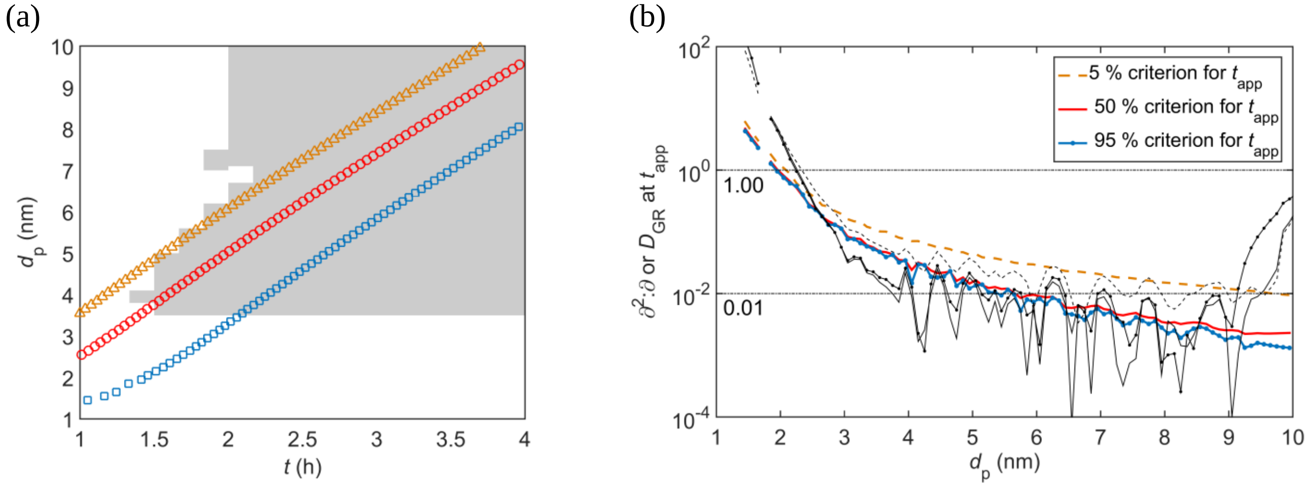

Supplementary Figure S 8: Panel (a): Region in the (time, size)-coordinate system where a continuous model is expected to be valid (∂^2:∂ less than 5 %; grey shade) for the LVOC model system at $C_{LVOC} = 2\cdot10^7$ cm^{-3}. Markers show the appearance times t_{app} of different particle sizes bins, with the appearance time defined as the moment at which the size bin reaches 5 % (triangles), 50 % (circles), or 95 % (squares) of its maximum concentration. Panel (b): Relative difference D_{GR} between GR_{app} and GR_{cond} (thin lines), and the metric ∂^2:∂ (thick lines) at t_{app}, with t_{app} defined as the moment at which the concentration in a given size bin reaches 5 % (dashed lines), 50 % (solid lines), or 95 % (solid lines with filled circles) of its maximum value.

2.4 Remarks on the interpretation of the apparent growth rate GR_{app}

GR_{app} determined from the simulation data exhibit a trend similar to previously reported experimental observations[1,11–13]: GR_{app} is low at a couple of nanometers, and shows a rather steep increase to higher values as the particle size increases. This type of behavior is often interpreted as a thermodynamic barrier hindering the growth at the smallest sizes. While evaporation definitely has a decreasing effect on particle growth rate, the time evolution of the size distribution at the smallest sizes, and consequently the apparent growth are not determined solely by deterministic condensation and evaporation processes. In addition to stochastics, due to their high mobility the smallest sizes are more strongly affected by sinks and coagulation processes compared to larger particles. Moreover, in the presence of a vapor source, the first particle sizes may appear when the vapor concentration is still increasing, further affecting the apparent size-dependent growth rate.

Supplementary Figure S 9 depicts the response of GR_{app} to changes in (1) the external conditions, namely particle sinks and vapor source (panel a), and (2) the thermodynamic properties of the nanoparticles (panel b) for LVOC. Results for the LVOC–ELVOC mixture are similar. Supplementary Figure S 9a demonstrates that the behavior of GR_{app} at the very first sizes is largely determined by particle sinks and

sources: a test simulation performed using a constant vapor concentration and omitting the external sink gives a different behavior for GR_{app} at the smallest sizes compared to the default laboratory chamber set-up including a vapor source and a particle sink onto the chamber walls (see also ref. 3). On the other hand, a simulation performed at chamber conditions with no Kelvin factor (the exponential factor in Eq. (S1)) results in a similar, increasing GR_{app} at the small sizes as simulations where the Kelvin factor is included (Supplementary Figure S 9b). This indicates that the existence of thermodynamic barriers in the growth of very small nanoparticles cannot be directly resolved based on the size dependence of the apparent growth rate.

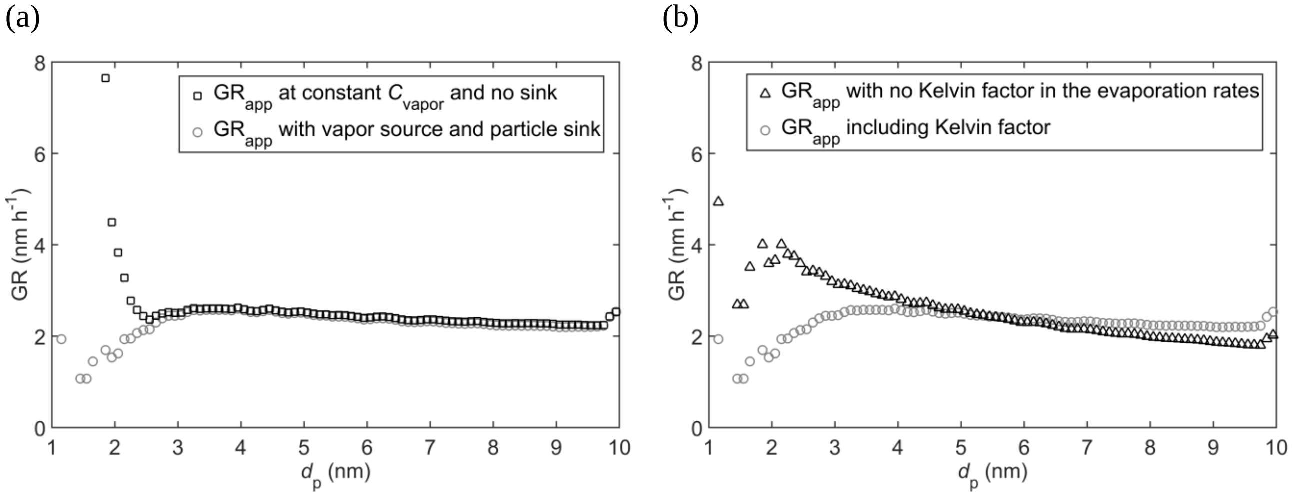

Supplementary Figure S 9: Panel (a): GR_{app} for a simulation of LVOC at a constant vapor concentration of $C_{vapor} = 2 \cdot 10^7$ cm^{-3} and no particle sinks, including the Kelvin factor in the evaporation rates. (The data points corresponding to the smallest size bins are ca. 30 nm h^{-1} or higher, and thus not shown for figure clarity.) Panel (b): GR_{app} for a simulation of LVOC with no Kelvin factor at laboratory chamber conditions with a vapor source of $Q = 2 \cdot 10^4$ cm^{-3} s^{-1} and a vapor and particle sink due to the chamber walls. Light circles show GR_{app} at chamber conditions with a vapor source of $Q = 2 \cdot 10^4$ cm^{-3} s^{-1}, corresponding to a final vapor concentration of $C_{vapor} = 2 \cdot 10^7$ cm^{-3}, including the Kelvin factor (Figure 1).

In general, vapor and particle sinks affect the apparent growth in different ways. First, sinks may affect ∂^2:∂ and D_{GR} by increasing the gradient of the particle size distribution function (Figure 2b). Second, sinks decrease particle life time, which shortens the time scale of steady-state equilibration and may lead to faster particle appearance and thus higher apparent growth[14]. Third, sinks affect the condensation and evaporation fluxes: scavenging decreases the concentrations of vapors and small clusters, decreasing the growth rate of a given particle. On the other hand, scavenging may also have an increasing effect on GR_{app} due to reduced concentrations of particles larger than the given size, as this reduction decreases the backward evaporation fluxes to the given particle[3].

2.5 Nanoparticle growth rates in atmospheric aerosol formation modeling

In regional and global climate and air quality models, the initial steps of aerosol particle formation from atmospheric vapors are commonly approximated by (1) determining the initial formation rate J ($d_{p,1}$) of nanoparticles of ca. $d_{p,1}$ = 1–1.5 nm based on nucleation theories, and (2) scaling the initial formation rate to a larger size, most often ca. $d_{p,2}$ = 3 nm, based on the estimated growth rate GR and particle scavenging sink S at sizes between $d_{p,1}$ and $d_{p,2}$ using the continuous GDE as[15,16]

$$J(d_{p,2}) = J(d_{p,1})\exp\left(-\int_{d_{p,1}}^{d_{p,2}} \frac{S}{GR} \mathrm{d}d_p\right). \qquad \text{(S6)}$$

The growth rate GR is typically assumed to be size-independent, and the loss rate S is approximated to decrease with increasing particle size as $S(d_p) = S_{ref} \cdot (d_p / d_{p,ref})^{-m}$, where S_{ref} is the loss rate at reference size $d_{p,ref}$, and m is a parameter normally set to −1.6 (Supplementary Table S 2). The reference size is often sulfuric acid vapor $d_{p,ref}$ = 0.71 nm[15]. With these assumptions, Eq. (S6) becomes

$$J(d_{p,2}) = J(d_{p,1})\exp\left[-\frac{S_{ref}}{GR}\frac{1}{(m+1)d_{p,ref}^{m}}\left(d_{p,2}^{m+1} - d_{p,1}^{m+1}\right)\right]. \qquad \text{(S7)}$$

While the continuous condensation approach is a source of uncertainty in Eqs. (S6) and (S7), the calculated J may be more severely distorted by the assumed growth rate GR. An overestimation of GR results in an overestimation of the formation rate $J(d_{p,2})$, with the quantitative error depending on the sink S_{ref} as well as the other parameters in the exponent of Eq. (S7). Assuming GR = 1 nm h^{-1}, using a typical boundary layer sink of $S_{ref} = 10^{-3}$ s^{-1}, and scaling J from 1 to 3 nm, an overprediction of a factor of 2 or 5 in GR leads to an increase of a factor of 2.3 or 3.8 in J , respectively. For $5 \cdot 10^{-3}$ s^{-1}, the corresponding values are 66 and 810. Generally, assuming GR between 1 and 5 nm h^{-1} and S_{ref} between 10^{-4} and 10^{-2} s^{-1} leads to an increase in J of a factor ranging from slightly more than one to over a hundred. Conditions where this error becomes particularly significant include relatively unpolluted environments with slow or average nanoparticle growth but non-negligible scavenging sink, as demonstrated by Supplementary Figure S 10.

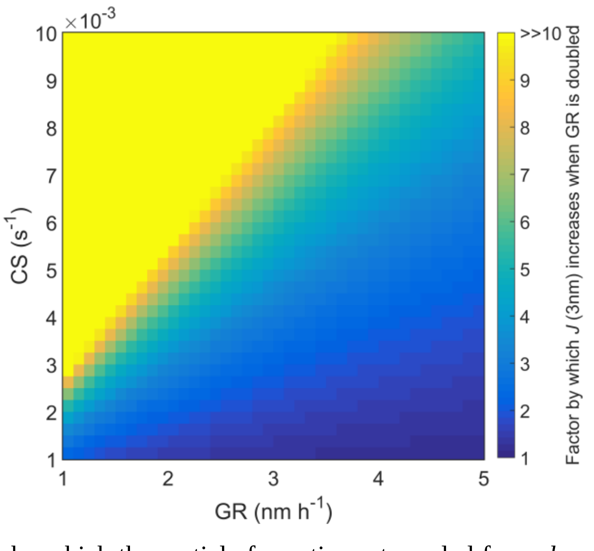

Supplementary Figure S 10: Factor by which the particle formation rate scaled from $d_{p,1}$ = 1 nm to $d_{p,2}$ = 3 nm by Eq. (S7) increases when the growth rate GR is overestimated by a factor of two for different values of GR and S_{ref}.

Supplementary references

1. Tröstl, J. *et al*. The role of low-volatility organic compounds in initial particle growth in the atmosphere. *Nature* **533**, 527-531 (2016).
2. Kurtén, T. *et al*. α-pinene autoxidation products may not have extremely low saturation vapor pressures despite high O:C ratios. *J. Phys. Chem. A* **120**, 2569-2582 (2016).
3. Olenius, T., Riipinen, I., Lehtipalo, K. & Vehkamäki, H. Growth rates of atmospheric molecular clusters based on appearance times and collision-evaporation fluxes: growth by monomers. *J. Aerosol Sci.* **78**, 55-70 (2014).
4. Elm, J. *et al*. Formation of atmospheric molecular clusters consisting of sulfuric acid and a $C_8H_{12}O_6$ tricarboxylic acid. *Phys. Chem. Chem. Phys.* **19**, 4877-4886 (2017).
5. Ortega, I. K. *et al*. From quantum chemical formation free energies to evaporation rates. *Atmos. Chem. Phys.* **12**, 225-235 (2012).
6. Almeida, J. *et al*. Molecular understanding of sulphuric acid-amine particle nucleation in the atmosphere. *Nature* **502**, 359-363 (2013).
7. Elm, J., Passananti, M., Kurtén, T. & Vehkamäki, H. Diamines can initiate new particle formation in the atmosphere. *J. Phys. Chem. A* **121**, 6155-6164 (2017).
8. Xie, H.-B. *et al*. Atmospheric fate of monoethanolamine: enhancing new particle formation of sulfuric acid as an important removal process. *Environ. Sci. Technol.* **51**, 8422-8431 (2017).
9. Gear, C. W. *Numerical initial value problems in ordinary differential equations* (Prentice-Hall, Englewood Cliffs, N. J., 1971).
10. Brown, P., Byrne, G. & Hindmarsh, A. VODE: a variable-coefficient ODE Solver. *SIAM J. Sci. and Stat. Comput.* **10**, 1038-1051 (1989).
11. Lehtipalo, K. *et al*. Methods for determining particle size distribution and growth rates between 1 and 3 nm using the Particle Size Magnifier. *Boreal Environ. Res.* **19**, 215-236 (2014).
12. Kulmala, M. *et al*. Direct observations of atmospheric aerosol nucleation. *Science* **339**, 943-946 (2013).
13. Bianchi, F. *et al*. New particle formation in the free troposphere: a question of chemistry and timing. *Science* **352**, 1109-1112 (2016).
14. Shi, G., Seinfeld, J. H. & Okuyama, K. Transient kinetics of nucleation. *Physical Review A* **41**, 2101-2108 (1990).
15. Lehtinen, K. E. J., Dal Maso, M., Kulmala, M. & Kerminen, V.-M. Estimating nucleation rates from apparent particle formation rates and vice versa: Revised formulation of the Kerminen-Kulmala equation. *J. Aerosol Sci.* **38**, 988-994 (2007).
16. Kerminen, V.-M. & Kulmala, M. Analytical formulae connecting the 'real' and the 'apparent' nucleation rate and the nuclei number concentration for atmospheric nucleation events. *J. Aerosol Sci.* **33**, 609-622 (2002).
17. Kürten, A., Williamson, C., Almeida, J., Kirkby, J. & Curtius, J. On the derivation of particle nucleation rates from experimental formation rates. *Atmos. Chem. Phys.* **15**, 4063-4075 (2015).
18. Olenius, T., Kupiainen-Määttä, O., Ortega, I. K., Kurtén, T. & Vehkamäki, H. Free energy barrier in the growth of sulfuric acid-ammonia and sulfuric acid-dimethylamine clusters. *J. Chem. Phys.* **139**, 084312 (2013).
19. Kürten, A. *et al*. Neutral molecular cluster formation of sulfuric acid-dimethylamine observed in real time under atmospheric conditions. *Proc. Natl. Acad. Sci. U. S. A.* **111**, 15019-15024 (2014).
